# Yeast lacking the sterol C-5 desaturase Erg3 are tolerant to the anti-inflammatory triterpenoid saponin escin

**DOI:** 10.1038/s41598-023-40308-0

**Published:** 2023-08-21

**Authors:** Emily J. Johnston, Jess Tallis, Edward Cunningham-Oakes, Tessa Moses, Simon J. Moore, Sarah Hosking, Susan J. Rosser

**Affiliations:** 1 Centre for Engineering Biology, University of Edinburgh, Edinburgh, EH9 3BD UK; 2 Department of Infection Biology and Microbiomes, Institute of Infection, Veterinary and Ecological Sciences, University of Liverpool, Liverpool, L69 7ZB UK; 3 EdinOmics, RRID:SCR_021838, University of Edinburgh, Max Born Crescent, Edinburgh, EH9 3BF UK; 4 Genetic Science Division, Thermo Fisher Scientific, 7 Kingsland Grange, Warrington, Cheshire, WA1 4SR UK; 5 Unilever R&D Port Sunlight, Quarry Road East, Bebington, Wirral, CH63 3JW UK

**Keywords:** Transcriptomics, Industrial microbiology, Metabolic engineering, Molecular engineering

## Abstract

Escin is a mixture of over 30 glycosylated triterpenoid (saponin) structures, extracted from the dried fruit of horse chestnuts. Escin is currently used as an anti-inflammatory, and has potential applications in the treatment of arthritis and cancer. Engineered yeast would enable production of specific bioactive components of escin at industrial scale, however many saponins have been shown to be toxic to yeast. Here we report that a *Saccharomyces cerevisiae* strain specifically lacking the sterol C-5 desaturase gene *ERG3*, exhibits striking enhanced tolerance to escin treatment. Transcriptome analyses, as well as pre-mixing of escin with sterols, support the hypothesis that escin interacts directly with ergosterol, but not as strongly with the altered sterols present in *erg3*Δ. A diverse range of saponins are of commercial interest, and this research highlights the value of screening lipidome mutants to identify appropriate hosts for engineering the industrial production of saponins.

Saponins (glycosylated triterpenoids) are amongst the most numerous and diverse groups of plant natural products, with huge potential in many industries^1,2^. In addition to pharmaceutical applications as vaccine adjuvants^3^ and anti-inflammatories^4^, saponins also have potential in therapeutics against arthritis, obesity, cancer and Alzheimer’s disease^5–7^. Some saponins have also been used as foam stabilisers and emulsifiers in cosmetics and soaps^8^. Many saponin compounds have haemolytic properties, and in nature saponins are generally considered to function as plant defence compounds, although few studies have investigated the degree and mechanism of antifungal activity^9–16^. This is of relevance towards understanding the biological role of saponins in plants, and towards the engineering of yeast as factories for saponin production. Whilst saponin extraction from plants is seasonal and of limited yield, yeast can easily be engineered to express plant genes, and there is established infrastructure for large-scale rapid yeast culture and product extraction. In contrast to bacteria, yeast have endoplasmic reticulum (ER) which support the activity of ER-localised cytochrome P450 enzymes that are involved in saponin biosynthesis. Production in engineered yeast also enables targeted saponin structures to be produced and extracted at high purity, in contrast to the mixtures of saponins which are extracted from plants.

Escin (also termed Aescin) is a mixture of over 30 saponin structures extracted from fruit of the horse chestnut tree *Aesculus hippocastanum* L. Approximately 60% of escin is β-escin (Fig. 1a), comprising a protoaescigenin triterpenoid backbone, esterified at the C-22 position with acetic acid, and at the C-21 position with angelic or tiglic acid^17^. Escin has potent anti-inflammatory properties, and is used in the treatment of chronic venous insufficiency, postoperative oedema and haemorrhoids^4,18,19^. Escin also has promise in the treatment of arthritis^20,21^ and cancer; escin interferes with tumour cell cycles, induces cancer cell apoptosis, inhibits cell migration, and increases the impact of chemotherapy drugs^22^. Whilst horse chestnut provides a seasonal supply of a mixture of saponins, engineered yeast could potentially enable year-round industrial-scale manufacture of specific active components of this potent therapeutic, however escin has previously been shown to have antifungal activity^23,24^.

**Figure 1:**
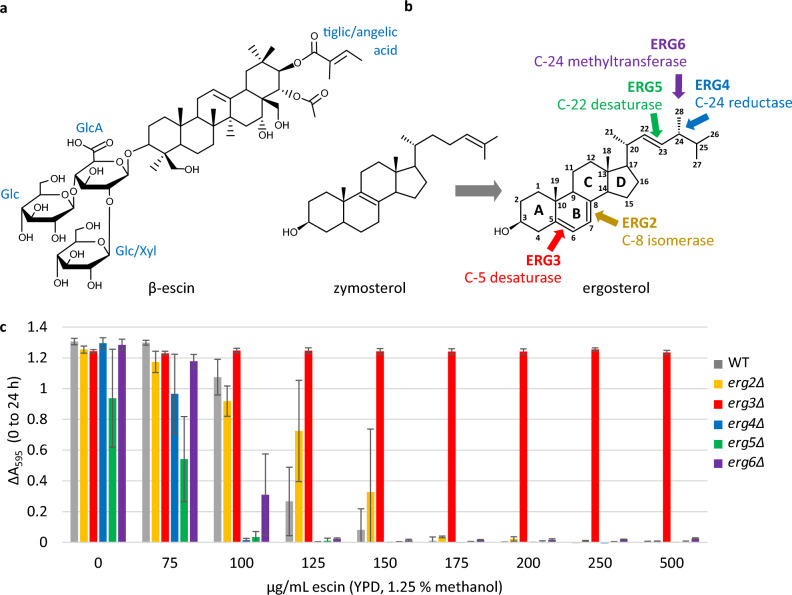
(**a**) Structure of β-escin, which comprises approximately 60% of saponins in escin^17^. The protoaescigenin backbone is esterified at C-22 with acetic acid and at C-21 with either angelic or tiglic acid (tiglic acid shown). Glucuronic acid (GlcA) is conjugated at C-3. Two glucose (Glc) sugars are attached to the glucuronic acid, one of which can be substituted with xylose (Xyl). (**b**) Activity of enzymes involved in the latter stages of ergosterol biosynthesis, from the precursor zymosterol. The reactions are depicted in Fig. S1. (**c**) Growth in microplate cultures indicated by ΔA_595_ (0–24 h). Three biological replicates ± standard deviation (SD).

Many saponins, including escin, have been reported to interact with the mammalian sterol cholesterol^9,17,25^. We hypothesised that escin may inhibit fungal growth via interaction with ergosterol, which comprises 12 mol% of the *S. cerevisiae* lipidome, and is the predominant sterol at the yeast plasma membrane^26,27^. In support of this, it has recently been shown that supplementation of *Leptosphaeria maculans* culture with ergosterol alleviates growth inhibition mediated by escin treatment^24^. We further hypothesised that yeast strains accumulating sterols other than ergosterol, may exhibit enhanced escin tolerance. Here we report that a *S. cerevisiae* strain lacking the sterol C-5 desaturase Erg3, exhibits striking enhanced tolerance to high concentrations of escin, supporting our hypotheses. Therefore, an engineered strain lacking Erg3 would enable microbial production of escin saponins.

## Results

### Yeast lacking the C-5 sterol desaturase Erg3 exhibit striking enhanced tolerance to escin

We hypothesised that if escin toxicity is mediated by direct interaction with ergosterol, then ergosterol biosynthesis mutants may differ in escin tolerance. Sterols are considered to have a key role in maintaining homeostasis of plasma membrane dynamics^28^, however yeast strains lacking enzymes which catalyse the final five steps in ergosterol biosynthesis (Erg2, Erg3, Erg4, Erg5 or Erg6; Figs. 1b and S1) are viable. Due to the substrate promiscuity of the latter enzymes in ergosterol biosynthesis, these deletion mutants accumulate a mixture of sterol structures, which differ from ergosterol in the number and position of double bonds in the sterol B-ring, and the sterol side chain^29^. We used microplate cultures of the wild type strain BY4741 (WT) and mutants from the Yeast Deletion Collection^30^, to assess growth of the ergosterol biosynthesis mutants in complex rich medium (YPD) in the presence and absence of escin (Fig. 1c). Sterol extraction and analysis by Gas Chromatography–Mass Spectrometry verified that ergosterol did not accumulate in the mutant strains (Figs. S2 and S3). The Minimum Inhibitory Concentration (MIC) of escin for the WT strain in YPD medium was 150 µg/mL. Similar MICs were observed for the *erg2*Δ, *erg4*Δ, *erg5*Δ and *erg6*Δ strains, however growth of the *erg3*Δ strain was uninhibited up to the highest concentration tested (1000 µg/mL; Fig. S4).

### Transcriptome analysis

In order to further explore the impact of escin on WT cells, and the mechanism of escin tolerance in *erg3*Δ, we analysed the transcriptomes of WT, *erg3*Δ and *erg6*Δ cells, treated with 0 or 100 µg/mL escin in YPD for 1 h, in shake flask cultures. The *erg6*Δ strain was included in this experiment as this strain shares many similar phenotypes with *erg3*Δ^29^, but does not have enhanced escin tolerance (MIC 150 µg/mL; Fig. 1c). Within the scope of this experiment, we also analysed the transcriptomes of *erg3*Δ and *erg6*Δ cells which were treated with 75 µg/mL escin for 1 h.

K means clustering of the 2000 genes with the most variable expression levels is shown in Fig. S5, with full details of gene set enrichment analysis included in the Supplementary Information (SI). Cluster B genes (n = 82) are in general expressed at a higher level in *erg3*Δ and *erg6*Δ compared to WT under all conditions and, as anticipated, this cluster is enriched in genes relating to sterol biosynthesis, sterol transport, siderophore transport, and regulation of transcription by glucose. Cluster A genes (n = 821) are downregulated in WT and *erg6*Δ in response to escin treatment. This cluster is enriched in genes relating to ribosome biogenesis and RNA processing. Cluster C (n = 897) and D (n = 200) genes are in general upregulated in WT and *erg6*Δ in response to escin treatment, with cluster D genes upregulated to a greater extent than cluster C. Cluster D is enriched in genes relating to trehalose, mannose, fructose and glutamate metabolism, as well as glycolysis and cell wall organisation. Cluster D is also enriched in genes associated with responses to osmotic, oxidative, temperature and starvation stress. Cluster C is enriched in genes relating to late nucleophagy, lipid catabolism, sulphur assimilation, the tricarboxylic acid cycle and gluconeogenesis.

**Figure 2:**
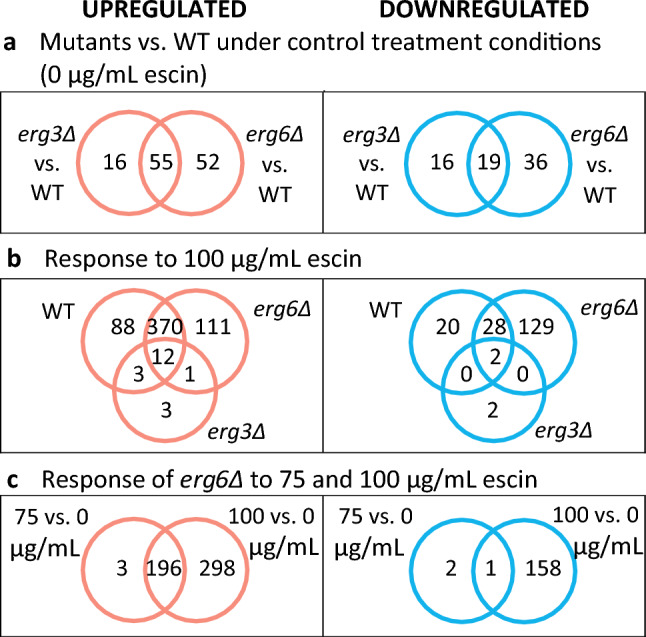
Venn diagrams for Differentially Expressed Genes (DEGs). DEGs have ≥ 2-fold differential expression, and a False Discovery Rate (FDR) ≤ 0.1.

Differentially Expressed Genes (DEGs) with ≥ 2-fold differential expression between conditions of interest (Fig. 2) are discussed in more detail below, with full gene set enrichment details in SI.

### Impact of *ERG3* and* ERG6* deletion on the yeast transcriptome

Comparing the transcriptomes of strains under control conditions reveals interesting differences between the mutant strains and WT. Deletion of *ERG6* results in a greater number of DEGs (n = 162) than *ERG3* deletion (n = 106). In both mutants, upregulated DEGs are enriched in genes relating to sterol biosynthesis and transport, including the sterol sensor and transcriptional activator *UPC2* (Fig. S6). In contrast, only a very small number of genes involved in sphingolipid and phospholipid supply are upregulated (*YSR3*, *RSB1*, *FAA2* and *ELO2* in both strains, and additionally *SUR1* in *erg6*Δ; Fig. S7), although large changes in sphingolipid composition have been reported for sterol biosynthesis mutants^31^.

**Figure 3:**
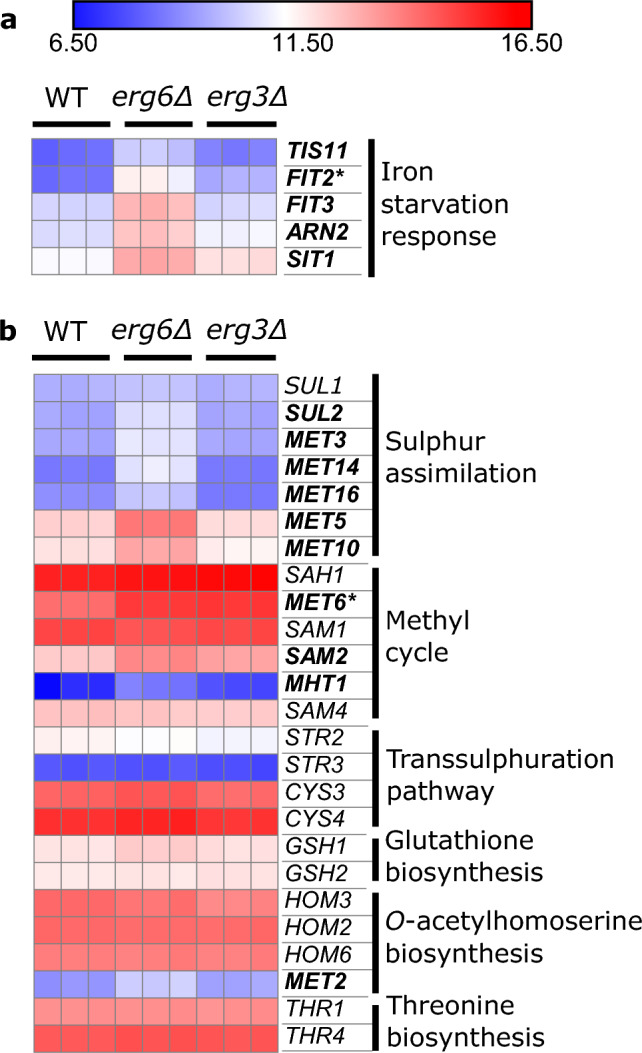
The *erg6*Δ strain exhibits elevated expression of sulphur assimilation genes and iron starvation response genes. Expression of genes relating to (**a**), iron homeostasis and (**b**), the methionine biosynthesis pathway, under control conditions, units log2(CPM + 4). Bold; gene upregulated ≥ 2-fold in *erg6*Δ versus WT. Asterisk; gene upregulated ≥ 2-fold in *erg3*Δ versus WT (FDR ≤ 0.1).

Anaerobic response cell wall mannoprotein genes are also highly upregulated in both strains (Fig. S8). Several steps of ergosterol biosynthesis require oxygen and iron as cofactors, and there is complex crosstalk between the regulatory responses to ergosterol, oxygen and iron^32–35^. A number of iron starvation response genes^36^ are upregulated in *erg6*Δ (*FIT2*, *FIT3*, *ARN2*, *SIT1*, *TIS11*), but not *erg3*Δ, relative to WT (Fig. 3a). It has recently been reported that the iron-sensing transcription factor Aft1, which usually shuttles between the cytoplasm and nucleus, accumulates in the vacuoles of ergosterol-diminished *upc2*Δ cells^37^. Our data suggests that this signalling pathway may be impacted to differing extents in *erg6*Δ and *erg3*Δ*.*

Methionine metabolism genes are also upregulated in *erg6*Δ, but not *erg3*Δ (Fig. 3b). Specifically, the upregulated genes are associated with the sulphur assimilation pathway (*SUL2*, *MET3*, *MET14*, M*ET16*, *MET5, MET10*), and the methyl cycle (*MET6*, *SAM2*, *MHT1*) which generates S-adenosylmethionine (SAM) (Fig. S9). The Erg6 enzyme uses the methyl donor SAM to methylate zymosterol^38^, and an Erg6*-*deficient strain has previously been found to accumulate SAM, which was attributed to reduced SAM consumption^39^.

**Figure 4:**
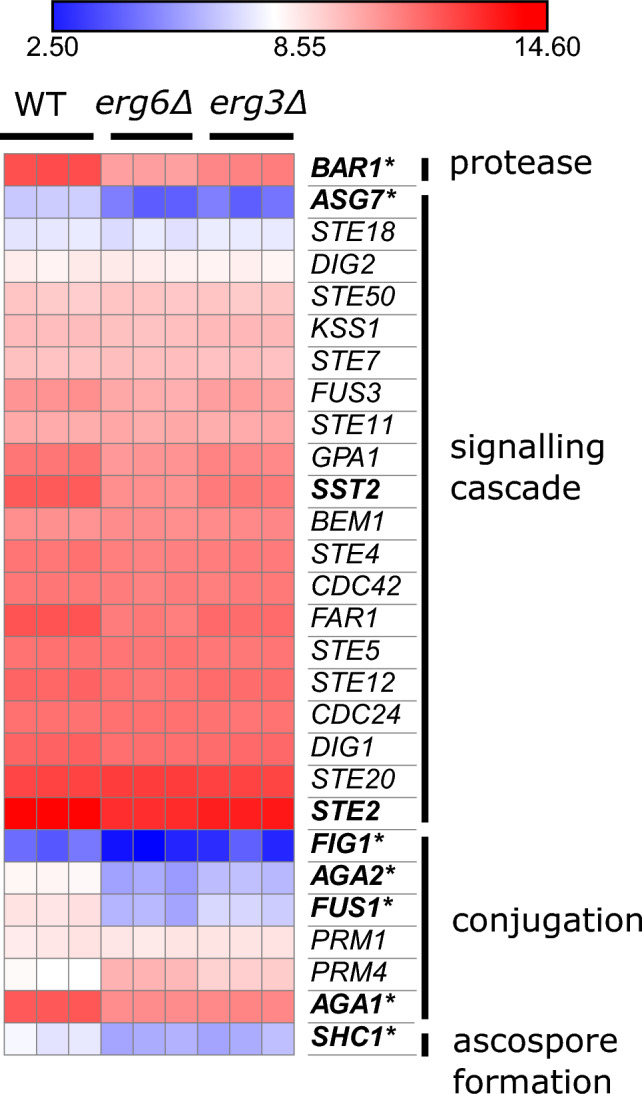
The *erg3*Δ and *erg6*Δ strains exhibit reduced expression of mating genes. Expression of genes relating to mating, units log2(CPM + 4). Bold; downregulated ≥ 2-fold in the comparison *erg6*Δ 0 µg/mL escin versus WT 0 µg/mL escin. Asterisk; downregulated ≥ 2-fold in the comparisons *erg3*Δ 0 µg/mL escin versus WT 0 µg/mL escin (FDR ≤ 0.1).

Both strains downregulate a number of genes involved in mating (Fig. 4). These are mainly genes which are involved in conjugation between mating schmoo tips (*FUS1*, *AGA1*, *AGA2*, *FIG1*, *SHC1*), and notably reduced mating efficiency has previously been described for ergosterol biosynthesis mutants^40,41^.

Both strains also express *NCE102* at a lower level than WT (42% of WT level in *erg3*Δ, and 40% of WT level in *erg6*Δ). Although primarily localised at plasma membrane eisosomes, Nce102 has recently been reported to have a role in vacuole fusion^42^, and vacuoles are highly fragmented in many ergosterol biosynthesis mutant strains, including *erg3*Δ, although not *erg6*Δ^43–45^.

It is notable that several DEGs between the mutants and WT are of unknown function; 20 and 29 upregulated DEGs, and 10 and 13 downregulated DEGs, in *erg3*Δ and *erg6*Δ respectively.

### Transcriptome responses to escin treatment

In response to 100 µg/mL escin, the WT and *erg6*Δ strains upregulate 473 and 494 genes, respectively (Fig. 2b). These DEGs are enriched in genes which are also associated with responses to desiccation, osmotic stress, oxidative stress, temperature, chemical treatment and changes in nutrient levels (SI). Treatment of *erg6*Δ cells with 75 µg/mL escin resulted in upregulation of 199 genes (Fig. 2c). These 199 DEGs are enriched in genes which are associated with responses to oxidative stress, chemical stress and starvation (SI).

The impact of escin treatment on central carbon metabolism genes is shown in Fig. S10. In response to 100 μg/mL escin, both WT and *erg6*Δ strongly upregulate genes relating to the biosynthesis of trehalose, glycogen and glycerol. The glucose polymer glycogen is considered to primarily function as a storage carbohydrate, which has little impact on the internal osmotic pressure of the cell^46,47^. The disaccharide trehalose is considered to have a key role in protecting the structure of lipid membranes and proteins, by displacing water from lipid bilayers and protein surfaces, and preventing aggregation of denatured proteins which would prevent their subsequent refolding^47^. Glycerol acts as a key osmolyte during hyperosmotic stress, and forms the backbone of phospholipids and storage lipid triacylglycerols^48^. The upregulation of trehalose, glycogen and glycerol biosynthesis is part of a general response to environmental change^47–49^. Additionally, both WT and *erg6*Δ strains upregulate genes encoding proteins of the Glucose Induced degradation Deficient (GID) complex, which negatively regulates gluconeogenesis, in favour of glycolysis, by initiating polyubiquitination and degradation of fructose-1,6-bisphosphatase^50^ (Fig. S11).

In response to 100 μg/mL escin, both WT and *erg6*Δ also upregulate genes encoding enzymes of the γ-aminobutyric acid (GABA) shunt pathway (Fig. S12) which degrades glutamate via GABA. In yeast, this pathway has been shown to be important for heat and oxidative stress tolerance^51^.

The upregulated DEGs are also enriched in genes relating to cell wall organisation, including sporulation and chitin biosynthesis (Fig. S13). Crosstalk between the High Osmolarity Glycerol 1 (Hog1) and Cell Wall Integrity (CWI) mitogen-activated protein kinase (MAPK) pathways mean that it is difficult to dissect specific stresses which are initiating the upregulation of these genes, without utilising mutants which are defective in specific branches of these signalling pathways^52–54^.

Many autophagy-related genes are upregulated in WT and *erg6*Δ in response to escin treatment (Fig. S14), including core phagosome components, and proteins involved in the cytoplasm-to-vacuole pathway, which selectively delivers hydrolases to the vacuole^55^. In response to 100 μg/mL escin, more autophagy-related genes are upregulated ≥ 2-fold in the *erg6*Δ strain than WT (11 vs. 7).

The *erg6*Δ strain also downregulates more genes than WT in response to 100 μg/mL escin (159 vs. 50), and these DEGs are enriched in genes relating to ribosome biogenesis and translation. These processes require substantial amounts of energy^56^, and their downregulation is part of a general stress response, in which resources are diverted to stress resistance rather than cell proliferation^57,58^.

If escin sequestered ergosterol from cells, or inhibited sterol sensing by interaction with either ergosterol or a sterol sensor, then we might expect to see a transcriptional change in sterol biosynthesis genes such as *ERG2* and *ERG11* in response to escin treatment. These genes are regulated by the sterol sensors and transcriptional activators Upc2 and Ecm22, in response to ergosterol content^59,60^. Differential expression of ergosterol biosynthesis genes is not observed (Fig. S6), although there is statistically significant but small upregulation of *UPC2* in each strain (1.3, 1.9 and 1.5-fold for WT, *erg6*Δ and *erg3*Δ respectively). A small number of genes related to sphingolipid supply are upregulated in response to escin in WT and *erg6*Δ (Fig. S7), although it should be noted that much of sphingolipid regulation is post-translational^31^.

In contrast to the large transcriptome changes observed for the WT and *erg6*Δ strains*,* the *erg3*Δ strain upregulates only 19 genes in response to 100 µg/mL escin, and downregulates 4 (Fig. S15). Most of these DEGs are also differentially expressed in the WT and/or *erg6*Δ strain in response to escin treatment, with the exception of *HAP4* (encoding a regulator of respiration), *YOL163W* (considered non-functional), *YGL088W* (unknown function) and the cell wall mannoprotein genes *DAN1* and *DAN4.*

### The *erg3*Δ transcriptome is not primed for the stress of escin treatment

In order to explore whether the escin response DEGs in the WT strain are already differentially expressed in *erg3*Δ under control conditions, potentially ‘priming’ *erg3*Δ for the stress of escin treatment, we compared DEGs in the comparisons WT 100 versus 0 µg/mL escin, and *erg3*Δ 0 µg/mL escin versus WT 0 µg/mL escin (Fig. S16a). Under control conditions, the *erg3*Δ strain has elevated expression of 18 genes which are also upregulated in WT in response to escin treatment, and lower expression of 5 genes which are downregulated in WT in response to escin treatment. A heatmap showing the expression levels of these overlapping genes is included in Fig. S16b. The majority of these genes are also differentially regulated in the *erg6*Δ strain under control conditions, and the *erg6*Δ strain does not exhibit the enhanced escin tolerance phenotype. The exceptions are *PAI3* (encoding a cytoplasmic proteinase A inhibitor), *SRL3* (encoding a cell cycle regulator), *HED1* (encoding a meiosis-specific protein), *MAL31* (encoding a maltose permease), *ARG3* (encoding ornithine carbamoyltransferase), *COS111* (encoding a mitochondrial protein), genes *YBR090C* and *YKR075C* of unknown function, and genes *YJR037W* and *YJL195C* which are unlikely to encode functional proteins.

Overall, this data shows that escin has no impact on growth and a negligible impact on the transcriptome of *erg3*Δ cells. This is not likely to be due to *erg3*Δ cells being already primed for the stresses induced by escin treatment, and is potentially attributable to the chemical composition of its membrane sterols.

### Pre-mixing escin with ergosterol, but not brassicasterol, prevents escin-mediated growth inhibition

In order to explore the hypothesis that escin mediates toxicity by directly interacting with ergosterol, but not the altered sterols present in *erg3*Δ, we assessed the impact of treating WT cells with escin alone, or escin which had been pre-mixed with ergosterol in a 1:1 molar ratio. It was considered that if ergosterol interacts directly with escin, then an escin-ergosterol mixture would have reduced impact on WT growth than escin alone, due to reduced accessibility of the escin saponins. Notably, yeast cells do not import exogenous sterols under aerobic conditions^37^.

The experiment was carried out using microplate cultures and complete supplement mixture (CSM) media, as opposed to YPD, as the exact content of CSM is defined, whereas YPD is a rich media containing complex molecules from yeast extract, which may also interact with escin and/or sterols. Previous studies have reported ergosta-7,22-dienol to be the predominant sterol in yeast lacking Erg3, with episterol and ergosta-7-enol also accumulating^31,43^. When sterol content was verified by Gas Chromatography-Mass Spectrometry (Figs. S2 and S3), the relative retention time of the largest sterol peak in the *erg3*Δ Total Ion Chromatogram corresponded to previous reports for ergosta-7,22-dienol, when the strain was grown in either YPD or CSM.

**Figure 5:**
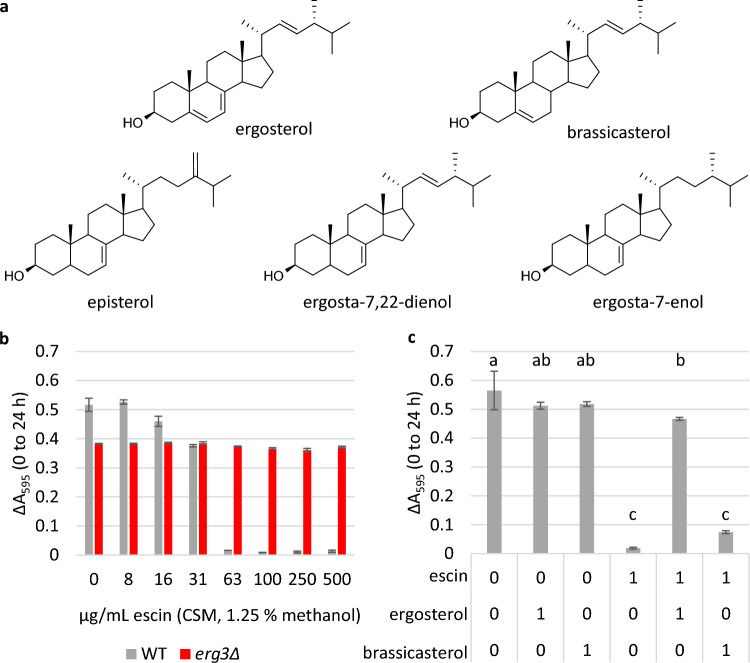
Restoration of growth when escin is pre-mixed with ergosterol, but not brassicasterol. (**a**) Structure of ergosterol (the predominant sterol in *S. cerevisiae* plasma membrane), brassicasterol (a plant sterol), and sterols which have been identified as accumulating in the *erg3*Δ strain (episterol, ergosta-7,22-dienol, ergosta-7-enol). (**b**) and (**c**), Growth in microplate cultures indicated by ΔA_595_ (0–24 h). Three biological replicates ± standard deviation (SD). In (**b**), the MIC of escin in CSM media is 63 μg/mL for WT, which is 2.4-fold lower than in rich complex medium. In (**c**), WT cells were grown in the presence of 0 or 63 μg/mL escin (1.25% methanol), alone or pre-mixed with ergosterol or brassicasterol in a 1:1 molar ratio. Ergosterol and brassicasterol only controls were included. Statistics: one-way ANOVA with post-hoc Tukey HSD test, conditions not connected by the same letter are significantly different (*p* ≤ 0.05).

The MIC of escin in CSM media was determined to be 63 μg/mL for WT cells (Fig. 5b); 2.4-fold lower than in YPD. Meanwhile growth of the *erg3*Δ strain was uninhibited up to the highest concentration tested (500 μg/mL). When escin was pre-mixed with ergosterol in a 1:1 molar ratio, and the mixture added to cells, growth of the WT strain in the presence of 63 μg/mL escin was completely restored (Fig. 5c). Pre-mixing escin with ergosterol also restored the percentage of cells stained with the membrane impermeable dye propidium iodide to control treatment levels (Fig. S17), indicating decreased cell permeability and/or increased cell viability.

The sterols present in *erg3*Δ are of limited availability, and so for comparison we also assessed growth when escin was pre-mixed with the phytosterol brassicasterol, which also has one double bond in the B-ring, although the position of this is between C-5 and C-6 (Fig. 5a). In contrast to the restored growth observed when escin was pre-mixed with ergosterol, restoration of WT growth was not observed when escin was pre-mixed with brassicasterol in a 1:1 molar ratio (Fig. 5c).

This supports the hypotheses that escin interacts directly with ergosterol, and that small changes in sterol structure can have a large impact on the strength of sterol-saponin interactions.

## Discussion

Saponins are a diverse group of high-value plant natural products, with huge potential in many industries. A recent increase in the identification of plant genes involved in saponin biosynthesis has enabled the engineering of yeast for saponin production^61^, however many saponins, including escin, are known to have antifungal activity and/or a lytic effect on red blood cells and synthetic membranes^9,23,25,62,63^. It is therefore likely that some saponins will inhibit yeast growth, particularly at high concentrations, which would limit productivity. Here, we explore the impact of the saponin mixture escin on yeast cells, as escin has potent therapeutic properties^4^, and production of specific escin saponins in yeast would facilitate cost-effective biosynthesis and purification of bioactive components, at industrial scale.

We report that the MIC of escin to WT yeast is 150 μg/mL in rich undefined (YPD) medium and 63 μg/mL in synthetic defined (CSM) medium. In addition to the richer YPD medium containing complex precursors to many biosynthetic processes, these media differ in carbon content, buffering capacity and osmolarity^64^. These differences are likely to contribute to the difference in MIC observed.

In order to investigate the hypothesis that escin inhibits yeast growth via interaction with ergosterol, we assessed the impact of escin on the growth of mutants in ergosterol biosynthesis. We identified that escin has negligible impact on growth of the sterol C-5 desaturase mutant *erg3*Δ*.* There was also little impact on the transcriptome of *erg3*Δ cells in response to escin treatment. In contrast there are large transcriptome changes in WT and *erg6*Δ cells in response to escin treatment, and the upregulated processes correspond to those induced in response to osmotic, oxidative, temperature and starvation stress. Due to cross-over between stress signalling pathways^52–54^, the specific initial perturbation which induces the transcriptome response cannot be pinpointed using transcriptome data alone. A comparison between genes which are differentially expressed in the WT strain in response to escin treatment, and genes which are differentially expressed in the *erg3*Δ strain compared to WT under control conditions, indicates that the *erg3*Δ transcriptome is not ‘primed’ for the stress of escin treatment. The finding that pre-mixing escin with ergosterol in a 1:1 molar ratio alleviates the growth inhibitory impact of escin to WT cells, supports the hypothesis that the toxicity of escin to yeast is mediated by direct interaction between escin saponins and ergosterol.

Phenotypes of the viable ergosterol biosynthesis mutants, and relevance to yeast cell factories, have recently been reviewed^29^. Mutants in ergosterol biosynthesis are usually more susceptible to antifungal treatment than WT cells^29^. This has been attributed to increased membrane permeability, hyperpolarisation of the plasma membrane, and reduced activity of efflux pumps such as Pdr5p in the mutant cells, resulting in increased intracellular accumulation of the antifungal drugs^29^. An exception to this is the enhanced fluconazole tolerance of *Candida albicans* strains lacking the *C. albicans* Erg3 enzyme^29^. Fluconazole inhibits the lanosterol 14-α-demethylase enzyme Erg11, resulting in accumulation of 14α-methylergosta8-24(28)dienol in cells with functional Erg3, and 14α-methyl-fecosterol in cells lacking Erg3 activity. It has been hypothesised that 14α-methylergosta8-24(28)dienol is detrimental to *C. albicans* cells^65^.

Ergosterol biosynthesis mutants also exhibit varying degrees of enhanced tolerance to the polyene antifungals nystatin and amphotericin B^29^. Nystatin binds ergosterol in synthetic liposomes, forming pores at high concentrations^66^, whilst amphotericin B sequesters ergosterol into cell surface aggregates^67^. It is plausible that the sterols present in the mutant strains do not interact as strongly with nystatin and amphotericin B as ergosterol does. Additionally, *S. cerevisiae erg6*Δ cells exhibit increased tolerance to the steroidal glycoalkaloid α-tomatine, whilst *erg3*Δ cells exhibit enhanced tolerance to tomatidine (the aglycone of α-tomatine)^68^. The steroidal glycoalkaloid α-tomatine has been reported to increase the permeability of synthetic vesicles via interaction with sterols^69^, and again, it is plausible that α-tomatine and tomatidine interact with the sterols present in *erg6*Δ and *erg3*Δ with lower affinity than ergosterol.

Recently, the impact of escin on synthetic model membranes has been studied using differential scanning calorimetry, wide-angle X-ray scattering, small-angle X-ray scattering and small angle neutron scattering. These studies indicate that escin increases the rigidity of 1,2-dimyristoyl-*sn*-glycero-3-phosphocholine (DMPC) phospholipid bilayers below the DMPC melting temperature, and increases the fluidity of DMPC bilayers above the DMPC melting temperature^17,62,63^. The membrane softening effect is likely due to headgroup interactions between the glycoside component of escin and the phosphocholine component of DMPC, whilst the stiffening effect is proposed to be due to incorporation of the rigid triterpenoid backbone of escin saponins into the lipid bilayer^17^. When cholesterol is included in DMPC bilayers, strong complexes form between cholesterol and escin saponins, reducing the amount of free escin available to interact with the phospholipid membrane, and resulting in deformation of small unilamellar vesicles^25^. It is plausible that escin saponins also interact with ergosterol, and similarly impact plasma membrane dynamics and organisation. This might induce pore formation, disrupt plasma membrane stability, and/or impact the lateral organisation and activity of plasma membrane lipids and proteins.

The finding that pre-mixing escin with ergosterol alleviates the inhibitory impact of escin, whilst pre-mixing with brassicasterol does not, and that *erg3*Δ mutants exhibit increased tolerance to escin treatment, whilst *erg2*Δ, *erg4*Δ, *erg5*Δ and *erg6*Δ do not, highlights that small changes in sterol and plausibly saponin structure can have a large impact on saponin bioactivity. The disruption of the C-5 desaturase gene *ERG3* is a promising engineering strategy for the production of escin saponins in engineered yeast. Strains with different lipidomes may exhibit enhanced tolerance to other saponin structures, and overall, this research demonstrates that screening the product tolerance of lipidome mutants should be a crucial prerequisite to the engineering of yeast strains for saponin production.

## Methods

### Yeast strains and media

This study used *Saccharomyces cerevisiae* BY4741 as the wild type background strain, and deletion mutants from the Yeast Deletion Collection^30^. YPD media; 10 g/L yeast extract, 20 g/L peptone, 20 g/L glucose. CSM media; 6.7 g/L yeast nitrogen base (Sigma Y0626), 790 mg/L Complete Supplement Mixture (Formedium DCS001), 20 g/L glucose.

### Microplate cultures for growth studies

Cells from overnight cultures were washed in sterile water and inoculated into 96 well plates (Thermo Scientific #167008), in 100 µL culture volumes, with starting OD_600_ 0.2 (equivalent to A_595_ 0.04 in the plate reader). Plates were incubated in Tecan Sunrise plate readers at 30 °C, with ‘normal’ shaking level. To assess growth A_595_ was recorded, which correlates with cell density.

### Extraction and analysis of yeast sterols

Yeast strains were inoculated into YPD or CSM media at OD_600_ 0.25 and incubated for 24 h (30 °C, 200 rpm). The cell density of each culture was then adjusted to OD_600_ 2.7, and approximately 36.9 × 10^6^ cells (from 1.5 mL of culture) were collected by centrifugation, and lysed in 500 µL lysis buffer (20% KOH, 50% ethanol, 80 nM coprostanol) for 10 min (100 °C). Sterols were extracted into 500 µL hexane three times. Organic phases were pooled, and 1 mL evaporated to dryness using a GeneVac EZ-2 Elite evaporator. Samples were trimethylsilylated with 50 µL pyridine:N-methylsilyltrifluoroacetamine (1:4) directly prior to Gas Chromatography Time-Of-Flight Mass Spectrometry (GC/QTOF-MS). Full GC/QTOF-MS details are included in SI.

### Transcriptome analysis

Overnight cultures in YPD media were used to inoculate 20 mL YPD in 100 mL conical flasks at OD_600_ 0.1. Cultures were grown (30 °C, 200 rpm shaking) to mid-log phase, and then treated with 0, 75 or 100 µg/mL escin in water for 60 min. Cell pellet samples of approximately 2 × 10^7^ cells were frozen in liquid nitrogen and stored at − 80 °C prior to RNA extraction. For RNA extraction, sequencing and data analysis methods, see Supplementary Information. RNA sequencing was carried out at the University of Liverpool Centre for Genomic Research.

### Propidium iodide (PI) assays

To prepare chambered slides (Ibidi 80807), 100 μL of 2 mg/mL concanavalin A (Sigma C2010) was pipetted into each well, incubated for 30 s, and aspirated off. Wells were then washed with water three times. Yeast was grown to OD_600_ 0.4 in CSM media, and 400 μL transferred to each well. After 5 min, the culture was aspirated off, wells were washed three times with treatment media (400 μL PBS 1.25% methanol, varying escin concentrations) and incubated for 1 h (30 °C, 200 rpm, dark). Cells were then washed three times with 400 μL of PBS containing 5 μg/mL PI (Invitrogen P3566), incubated for 20 min (30 °C, 200 rpm, dark), washed three times with 400 μL PBS and imaged on a Leica DMi8 inverted microscope (540-580/592-668 ex/em).

### Supplementary Information

Supplementary Information 1.

Supplementary Information 2.

Supplementary Information 3.

Supplementary Information 4.

## Data Availability

Supplementary figures, methods and details of Differentially Expressed Genes and Gene Set Enrichment results are included in supplementary files. RNA sequencing data is available at the European Nucleotide Archive (Project Accession Number PRJEB61952).
