# Supplementary material for: Yeast lacking the sterol C-5 desaturase Erg3 are tolerant to the anti-inflammatory triterpenoid saponin escin
Source: Sci Rep. 2023 Aug 21;13:13617. doi: 10.1038/s41598-023-40308-0 (PMC10442444; doi:10.1038/s41598-023-40308-0)
Supplement: Supplementary file 1 — Supplementary Information 1. [file 41598_2023_40308_MOESM1_ESM.pdf]

Yeast lacking the sterol C-5 desaturase Erg3 are tolerant to the anti-inflammatory triterpenoid saponin escin

Authors

Emily J. Johnston[1*], Jess Tallis[1], Edward Cunningham-Oakes[2], Tessa Moses[3], Simon J. Moore[4], Sarah Hosking[5], Susan J. Rosser[1*]

Author affiliations

[1]Centre for Engineering Biology, University of Edinburgh, Edinburgh, EH9 3BD, UK
[2]Department of Infection Biology and Microbiomes, Institute of Infection, Veterinary and Ecological Sciences, University of Liverpool, Liverpool, L69 7ZB, UK
[3]EdinOmics, RRID:SCR_021838, University of Edinburgh, Max Born Crescent, Edinburgh EH9 3BF, UK
[4]Genetic Science Division, Thermo Fisher Scientific, 7 Kingsland Grange, Warrington, Cheshire, WA1 4SR, UK
[5]Unilever R&D Port Sunlight, Quarry Road East, Bebington, Wirral, CH63 3JW, UK

***Contact information**

Emily J. Johnston: emily.johnston@ed.ac.uk.
Susan J. Rosser: susan.rosser@ed.ac.uk

Supplementary Information

Methods

Gas Chromatography Time-Of-Flight Mass Spectrometry (GC/QTOF-MS)

GC/QTOF-MS was conducted using an Agilent 7200B GC/Q-TOF mass spectrometer with Gerstel Multi-Purpose Sampler (MPS) robotics (Anatune). Samples were derivatised directly prior to GC/QTOF-MS using the MPS; to each vial, 50 µL of pyridine:N-methylsilyltrifluoroacetamine (1:4) was added, vortexed (30 seconds), incubated (30 min, 37 °C, 750 rpm), and then centrifuged (2 min, 4,500 rpm). Trimethysilylated samples of 1 µL were injected with a 1:10 split ratio into a DB-5ms 30 m × 250 µm × 0.25 µm GC column (Agilent Technologies). Helium was used as the carrier gas at a flow rate of 1 mL/min. The inlet was set to 250 °C, and the GC oven was programmed to hold at 100 °C for 2 min, then increase to 300 °C (at 25 °C/min), where it was held for 25 min. The ion source was set to 230 °C, with 35 µA filament current and 70 eV electron energy. A mass range of 35-600 *m/z* was scanned at an acquisition rate of 5 spectra/s with a solvent delay of 13 min.

RNA extraction and sequencing

Cell pellets of approximately 2×10^7 cells were lysed using 100 U of lyticase (Sigma L2524), and RNA was extracted using RNeasy Mini Kit (QIAGEN 74104) following the manufacturer's instructions, with on-column DNase treatment (RNase-free DNase set; QIAGEN 79254). The RNA was eluted into 50 µL DEPC-treated water (Ambion AM9915G).

cDNA synthesis

500 ng of RNA was reverse transcribed to cDNA using SuperScript IV (Invitrogen 18091200) and Oligo $d(T)_{20}$ primers according to manufacturer's instructions, with RiboLock RNase Inhibitor (ThermoScientific EO0381) and Ribonuclease H (Invitrogen 18021071).

RNA sequencing

At the University of Liverpool Centre for Genomic Research, total RNA samples were treated concomitantly with DNase (Turbo DNA-*free*™ kit; AM1907; Ambion by Life Technologies) and SUPERase•In RNase Inhibitor (AM2694; Invitrogen). After treatment, the samples were purified by use of Agencourt RNAClean XP beads (A66882; Beckman-Coulter). rRNA depletion was carried out by use of the NEBNext® rRNA Depletion Kit (E7850L; New England BioLabs), but substituting the bacterial baits with yeast baits. NEBNext Ultra II Directional RNA libraries were prepared from rRNA depleted RNA (E7760L; New England BioLabs). Paired-end RNA-Seq reads were generated using an Illumina NovaSeq6000.

Analysis of RNA sequencing data

First, raw FASTQ files were trimmed for the presence of Illumina adapter sequences (wherever the 3' end matched the adapter sequence for ≥3 bp) using Cutadapt (v1.2.1). Reads were further trimmed using Sickle (v1.200), with a minimum window quality score of 20, and reads <10 bp after trimming were removed. Additional read quality checks were performed using FastQC (v0.11.9) and MultiQC (v1.10). Reads were then mapped to a representative genome annotation for BY4741 (http://sgd-

archive.yeastgenome.org/sequence/strains/BY4741/BY4741_Toronto_2012/) from the Saccharomyces Genome Database, using the universal RNA-sequence aligner, STAR (v2.7.7a). Resultant BAM files were quality-filtered, using a mapping quality (MAPQ) cut-off of 244. Quality filtered files were then assessed for quality using the depth utility of Samtools (v1.9). Quality-assessed BAM files were then used with the genome annotation for BY4741, to generate a table of aligned read counts, using featureCounts (v2.0.1) from the subread package, with the minimum number of overlapping bases in a read required for read assignment set to 150 bp. Analysis of Differentially Expressed Genes was conducted using iDEP (v9.2)[1]. Gene Set Enrichment analysis was carried out using ShinyGO (v0.61)[2]. Heatmaps for selected gene sets were generated using Morpheus (https://software.broadinstitute.org/morpheus).

Tables

Figures

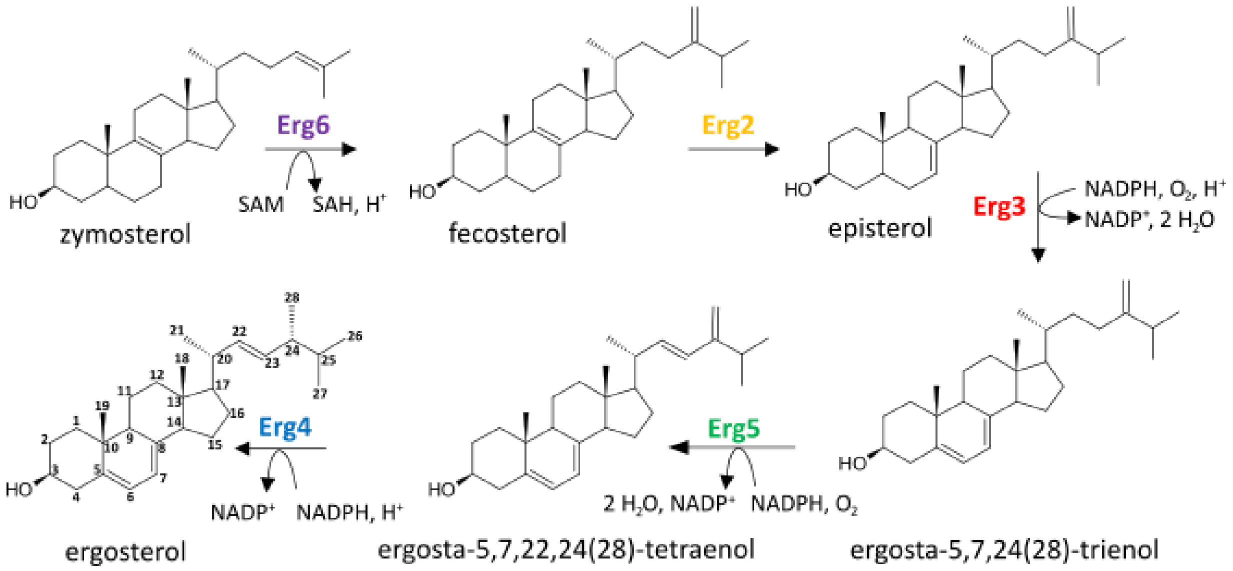

Figure S1: Reactions catalysed in the late steps of ergosterol biosynthesis

The five reactions in the conversion of zymosterol to ergosterol are often depicted sequentially as shown here, however the enzymes involved exhibit some promiscuity in sterol substrate. Erg6 is a C-24 sterol methyltransferase. Erg2 is a C-8 sterol isomerase. Erg3 is a sterol C-5 desaturase. Erg5 is a C-22 sterol desaturase. Erg4 is a C-24 sterol reductase. SAM, S-adenosyl-L-methionine. SAH, S-adenosyl-L-homocysteine.

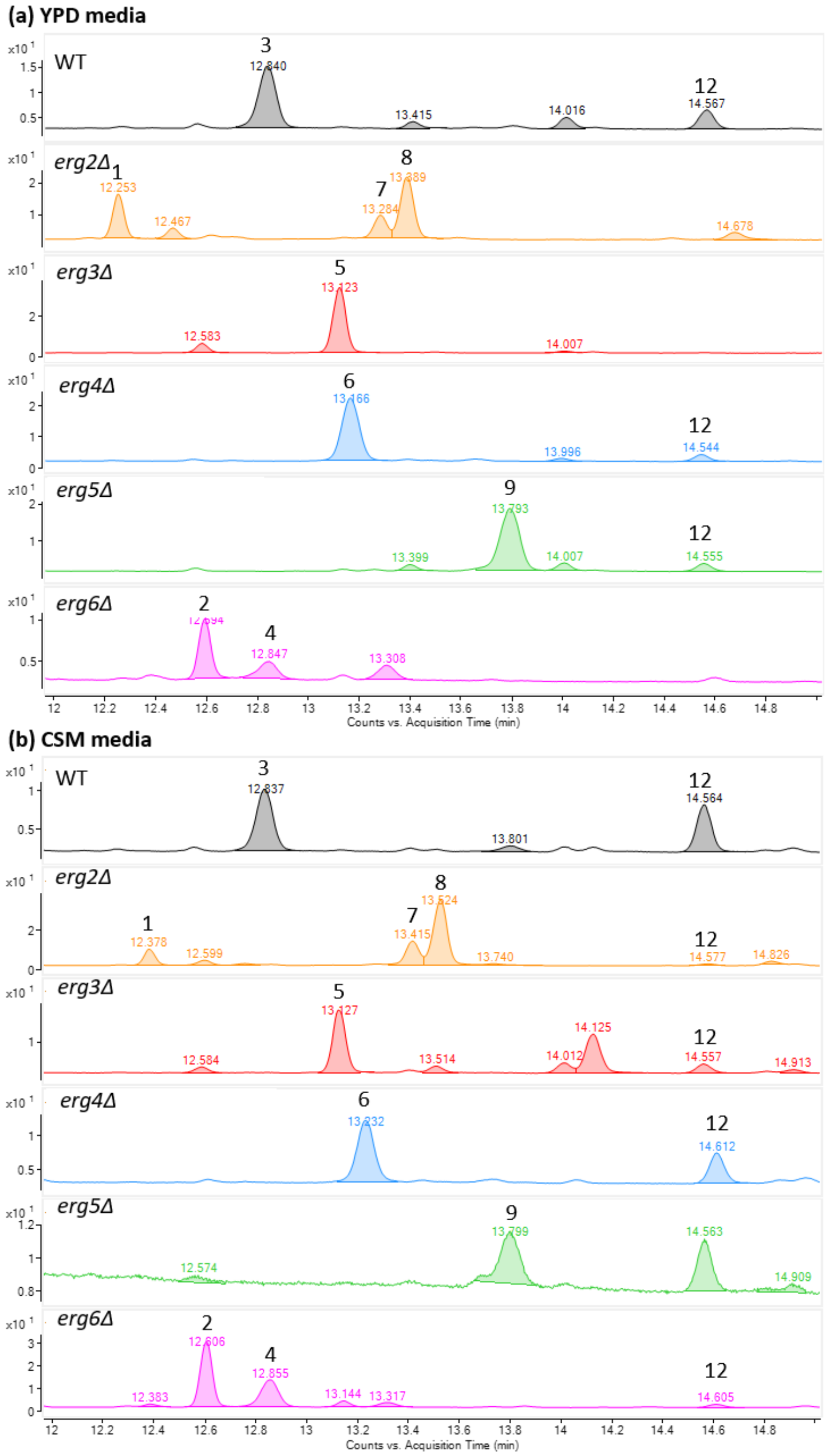

Figure S2: Total Ion Chromatograms for sterol extracts

Total Ion Chromatograms (TICs) showing peaks corresponding to sterols extracted from yeast strains cultured in either (a) YPD or (b) CSM media for 24 hours. Peak annotation based on ion m/z, relative retention times and relative abundances which have been previously reported[3–6]: (1) ergosta-5,8,22-trienol, (2) cholesta-8,24(25)-dienol (zymosterol), (3) ergosterol, (4) cholesta-5,7,24(25)-trienol, (5) ergosta-7,22-dienol, (6) ergosta-5,7,22,24(28)-tetraenol, (7) ergosta-8,24(28)-trienol (fecosterol), (8) ergosta-8-enol, (9) ergosta-5,7-dienol, (10) ergosta-7,24(28)-dienol (episterol), (11) ergosta-7-enol and (12) lanosterol. Ergosterol and lanosterol were verified using reference standards.

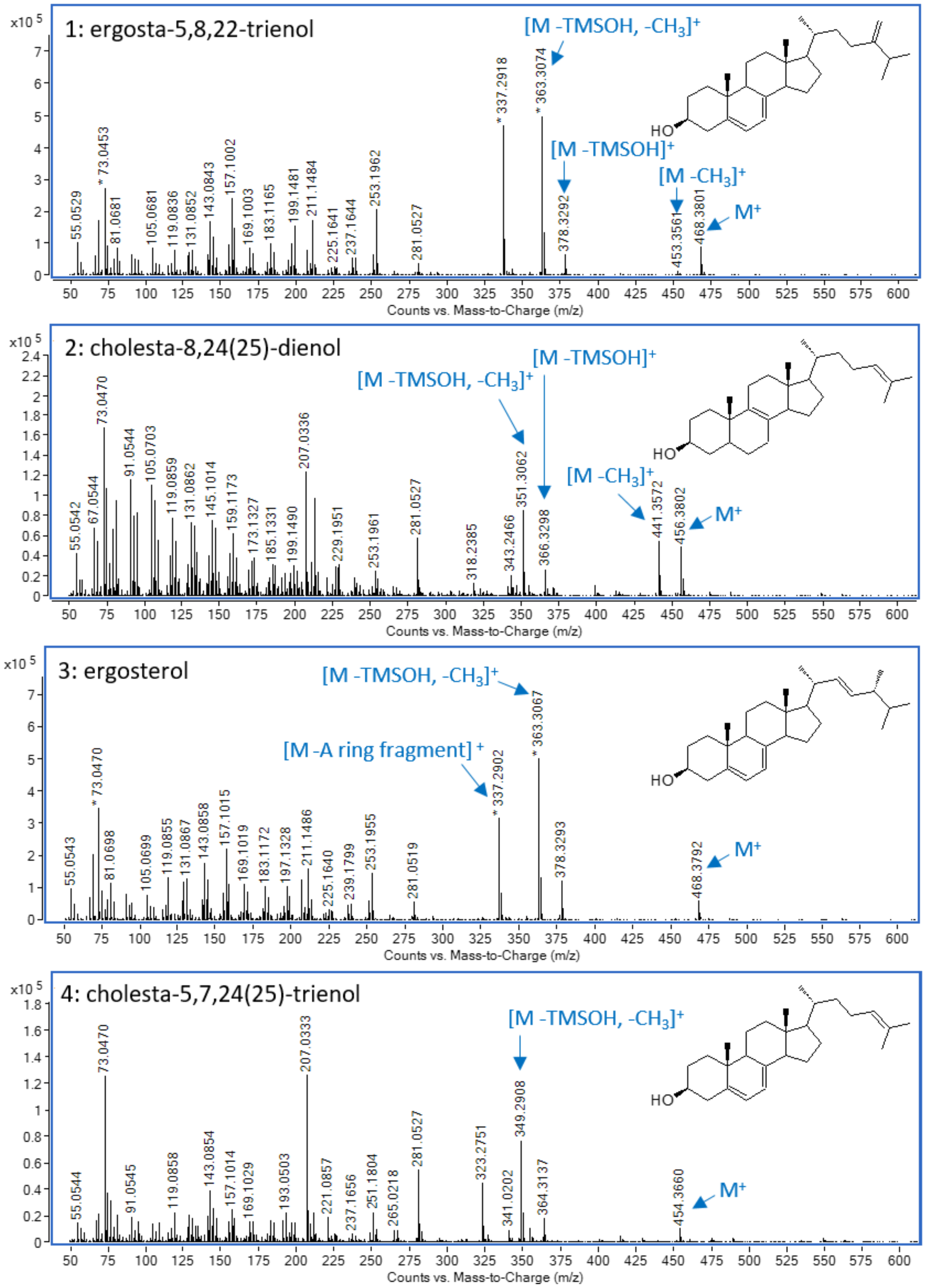

1: ergosta-5,8,22-trienol
[M -TMSOH, -CH$_3$]$^+$
[M -TMSOH]$^+$
[M -CH$_3$]$^+$
M$^+$
HO
55.0529
* 73.0453
81.0681
105.0681
119.0836
131.0852
143.0843
157.1002
169.1003
183.1165
199.1481
211.1484
225.1641
237.1644
253.1962
281.0527
* 337.2918
* 363.3074
378.3292
453.3561
468.3801
2: cholesta-8,24(25)-dienol
[M -TMSOH]$^+$
[M -TMSOH, -CH$_3$]$^+$
[M -CH$_3$]$^+$
M$^+$
HO
55.0542
67.0544
73.0470
91.0544
105.0703
119.0859
131.0862
145.1014
159.1173
173.1327
185.1331
199.1490
207.0336
229.1951
253.1961
281.0527
318.2385
343.2466
351.3062
366.3298
441.3572
456.3802
3: ergosterol
[M -TMSOH, -CH$_3$]$^+$
[M -A ring fragment]$^+$
M$^+$
HO
55.0543
* 73.0470
81.0698
105.0699
119.0855
131.0867
143.0858
157.1015
169.1019
183.1172
197.1328
211.1486
225.1640
239.1799
253.1955
281.0519
* 337.2902
* 363.3067
378.3293
468.3792
4: cholesta-5,7,24(25)-trienol
[M -TMSOH, -CH$_3$]$^+$
M$^+$
HO
55.0544
73.0470
91.0545
119.0858
143.0854
157.1014
169.1029
193.0503
207.0333
221.0857
237.1656
251.1804
265.0218
281.0527
323.2751
341.0202
349.2908
364.3137
454.3660
x10 5
Counts vs. Mass-to-Charge (m/z)

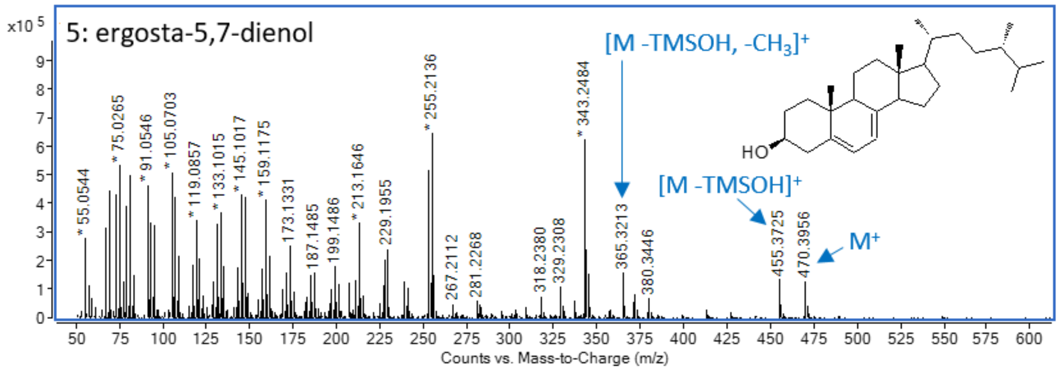
5: ergosta-5,7-dienol
x10 5
[M -TMSOH, -CH3]+
[M -TMSOH]+
M+
HO
* 55.0544
* 75.0265
* 91.0546
* 105.0703
* 119.0857
* 133.1015
* 145.1017
* 159.1175
173.1331
187.1485
199.1486
* 213.1646
229.1955
* 255.2136
267.2112
281.2268
318.2380
329.2308
* 343.2484
365.3213
380.3446
455.3725
470.3956
Counts vs. Mass-to-Charge (m/z)

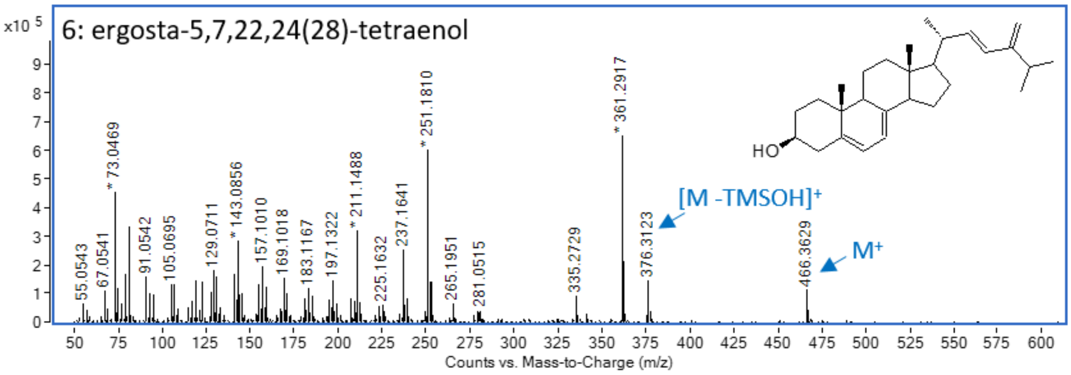
6: ergosta-5,7,22,24(28)-tetraenol
x10 5
[M -TMSOH]+
M+
HO
55.0543
67.0541
* 73.0469
91.0542
105.0695
129.0711
* 143.0856
157.1010
169.1018
183.1167
197.1322
* 211.1488
225.1632
237.1641
* 251.1810
265.1951
281.0515
335.2729
* 361.2917
376.3123
466.3629
Counts vs. Mass-to-Charge (m/z)

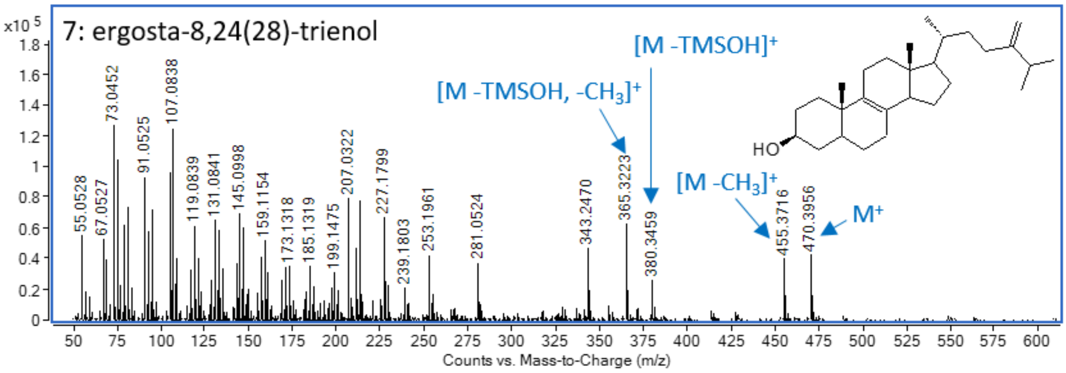
7: ergosta-8,24(28)-trienol
x10 5
[M -TMSOH]+
[M -TMSOH, -CH3]+
[M -CH3]+
M+
HO
55.0528
67.0527
73.0452
91.0525
107.0838
119.0839
131.0841
145.0998
159.1154
173.1318
185.1319
199.1475
207.0322
227.1799
239.1803
253.1961
281.0524
343.2470
365.3223
380.3459
455.3716
470.3956
Counts vs. Mass-to-Charge (m/z)

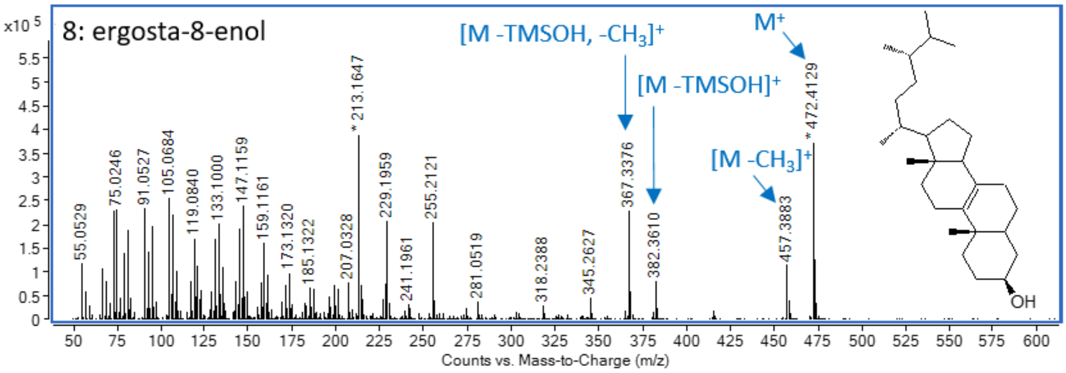
8: ergosta-8-enol
x10 5
[M -TMSOH, -CH3]+
M+
[M -TMSOH]+
[M -CH3]+
OH
55.0529
75.0246
91.0527
105.0684
119.0840
133.1000
147.1159
159.1161
173.1320
185.1322
207.0328
* 213.1647
229.1959
241.1961
255.2121
281.0519
318.2388
345.2627
367.3376
382.3610
457.3883
* 472.4129
Counts vs. Mass-to-Charge (m/z)

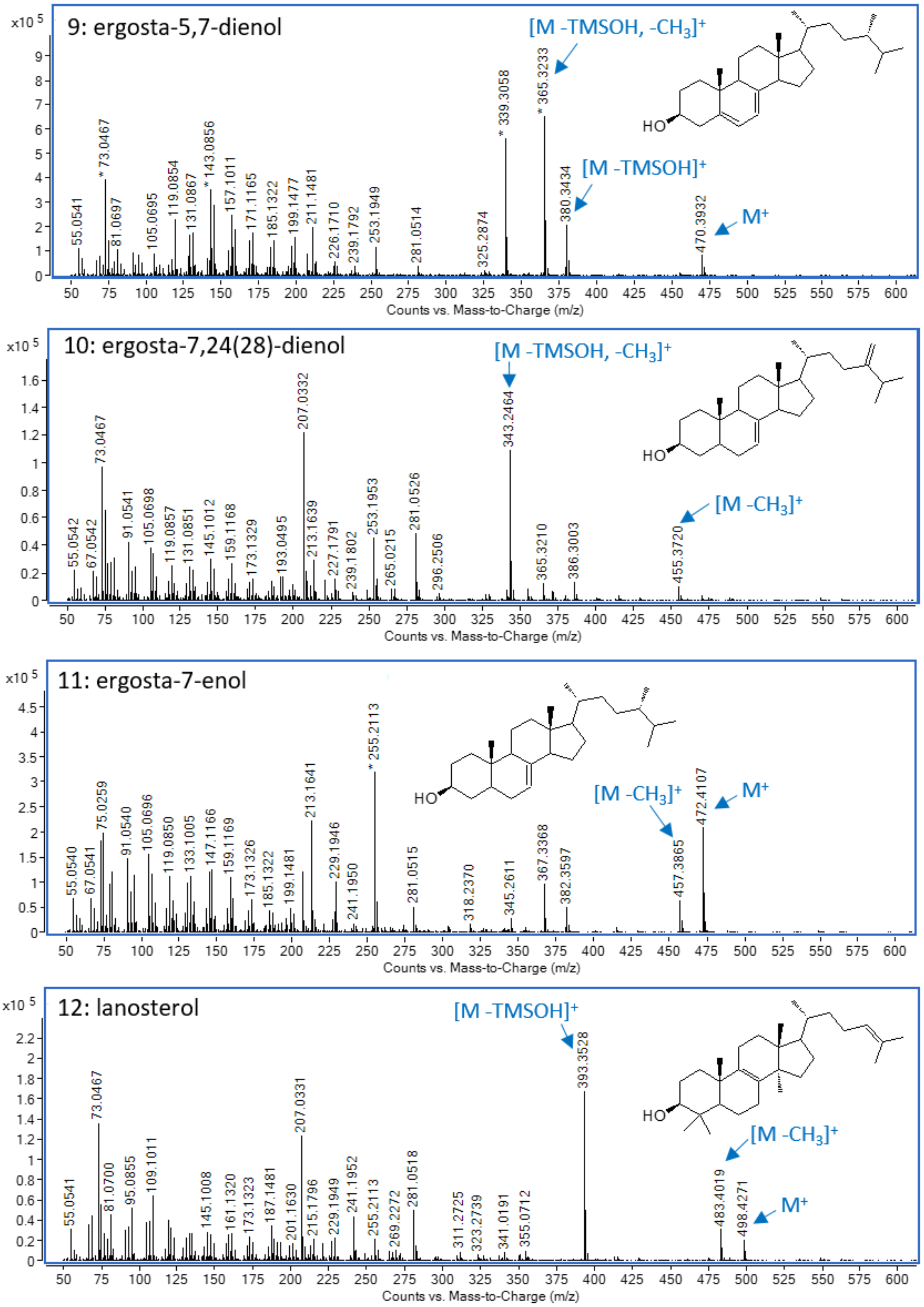

Figure S3: Sterol fragmentation patterns

Ions at the TIC peaks annotated 1 to 12 in Figure S2. M, parent ion with trimethylsilylation of the hydroxyl groups. TMSOH, trimethylsilanol. The characteristic ergosterol fragment ion at m/z 337 results from fragmentation of the A ring with loss of carbon atoms 1 to 3 and their substituents, and transfer of hydrogen from C-9[7]. Except for ergosterol and lanosterol which were verified using reference standards, sterol annotation is speculative based on ion m/z, relative retention times and abundances which have been previously reported[3–6].

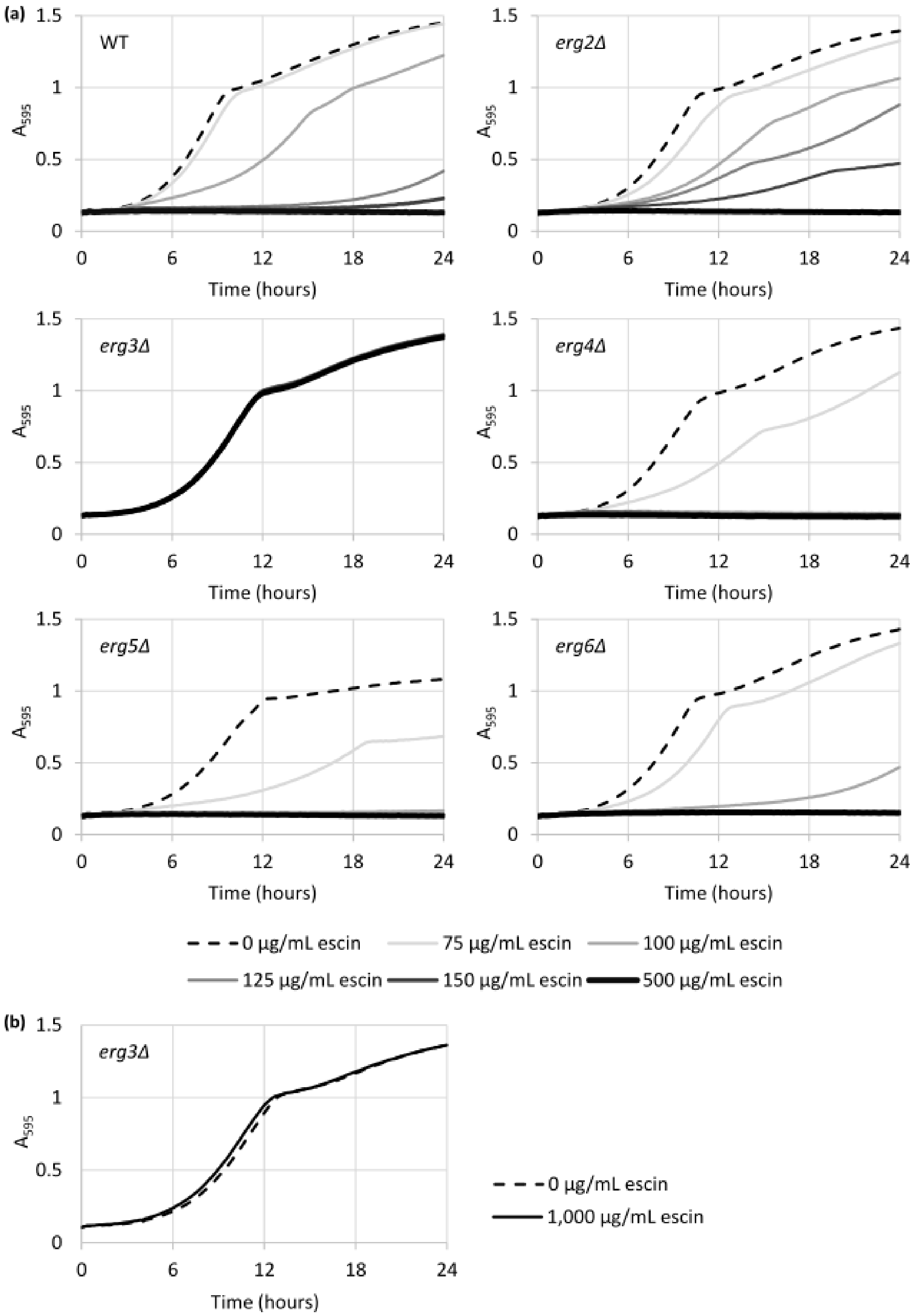

Figure S4: Growth of ergosterol biosynthesis mutants in the presence of escin
a, Growth of microplate cultures indicated by A_{595}, in the presence of different concentrations of escin. YPD media 1.25 % methanol. Average of three biological replicates shown. **b**, Growth of the *erg3Δ* strain in the presence of 0 and 1,000 µg/mL escin, YPD media 2.5 % methanol. Average of three biological replicates.

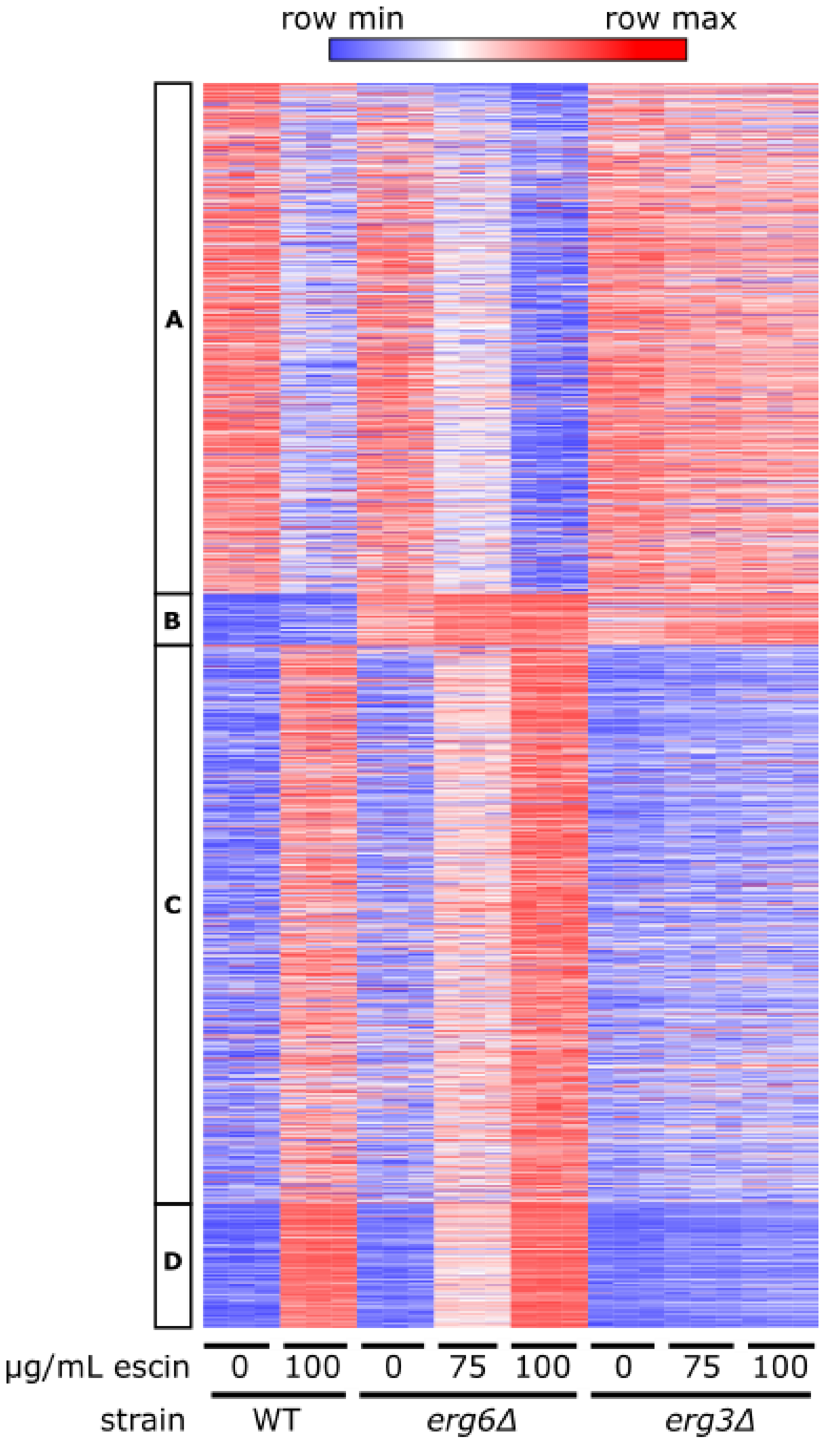

Figure S5: K means clustering of the 2,000 most variable genes
Expression as log2(CPM+4). Cluster A (n= 821) is enriched in genes relating to ribosome biogenesis and RNA processing. Cluster B (n= 82) is enriched in genes relating to sterol biosynthesis, sterol transport, siderophore transport, and regulation of transcription by glucose. Cluster C (n= 897) is enriched in genes relating to late nucleophagy, lipid catabolism, sulphur assimilation, the tricarboxylic acid cycle and gluconeogenesis. Cluster D (n= 200) is enriched in genes relating to trehalose, mannose, fructose and glutamate metabolism, glycolysis and cell wall organisation. Cluster D is also enriched in genes associated with responses to osmotic, oxidative, temperature and starvation stress.

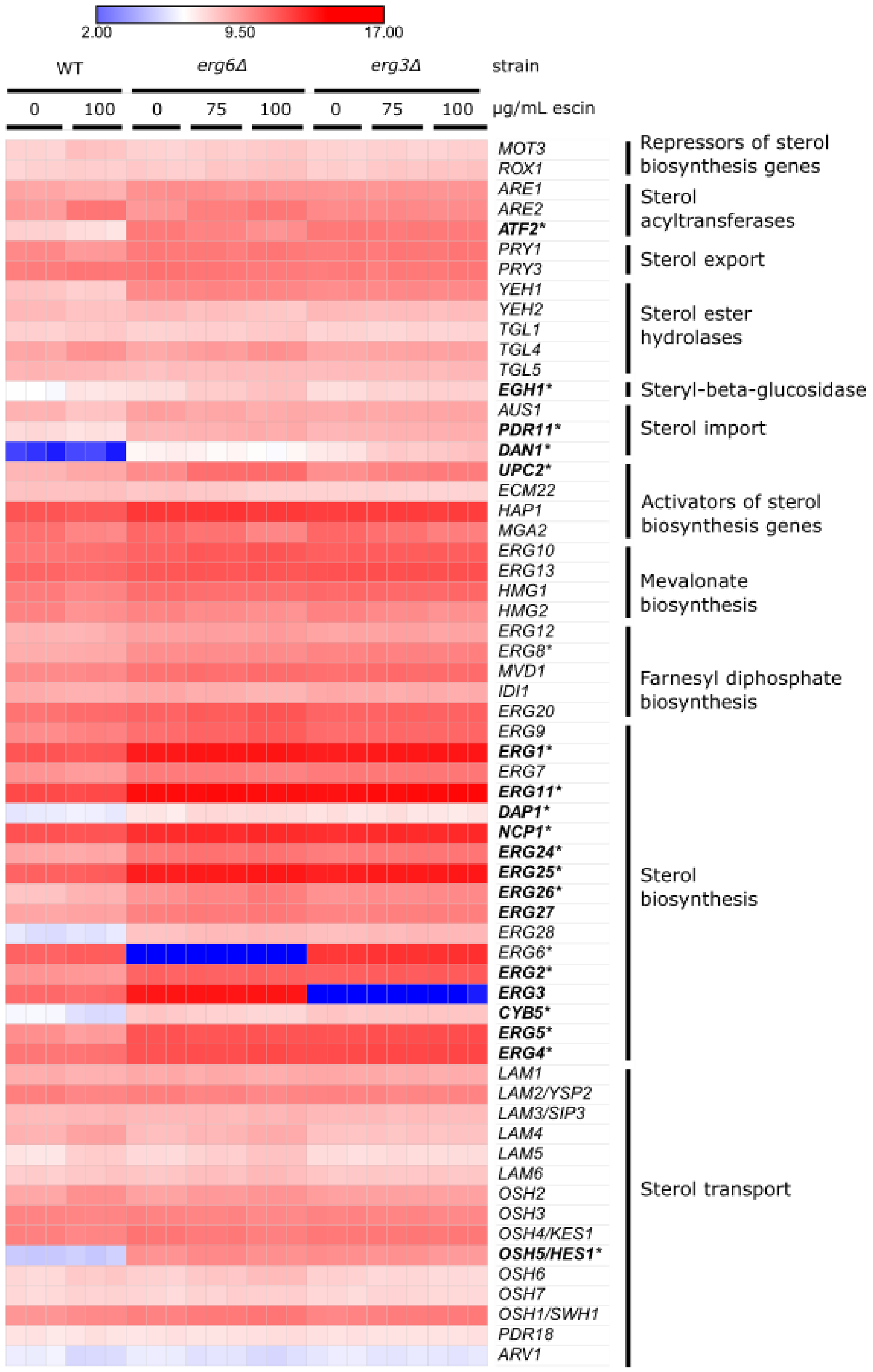

Figure S6: Expression of genes relating to sterol biosynthesis and transport
Expression scale log2(CPM+4). Bold; upregulated ≥2-fold in the comparison *erg6Δ* vs. WT (0 µg/mL escin). Asterisk; upregulated ≥2-fold in the comparisons *erg3Δ* vs. WT (0 µg/mL escin). Comparison FDR ≤0.1.

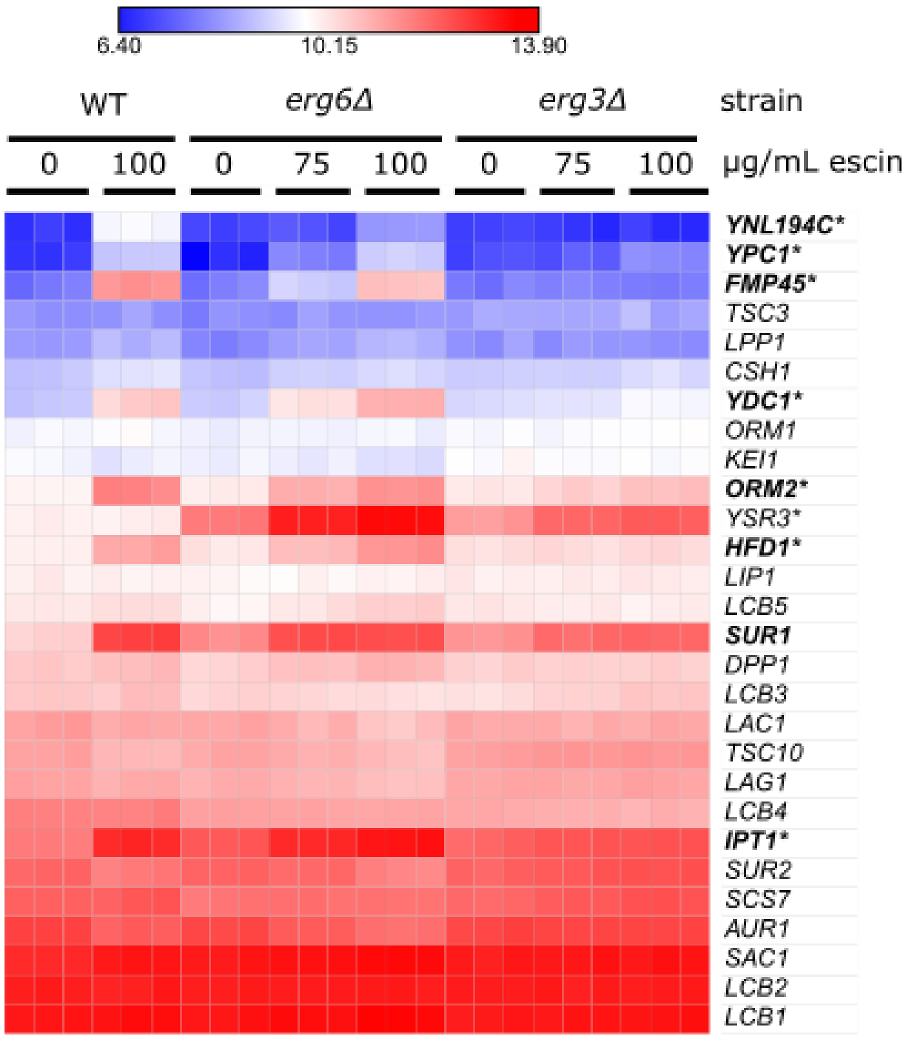

Figure S7: Expression of genes relating to sphingolipid supply
Expression scale log2(CPM+4). Bold; upregulated ≥2-fold in the comparison WT 100 vs. 0 μg/mL escin. Asterisk; upregulated ≥2-fold in the comparisons *erg6Δ* 100 vs. 0 μg/mL escin. Comparison FDR ≤0.1.

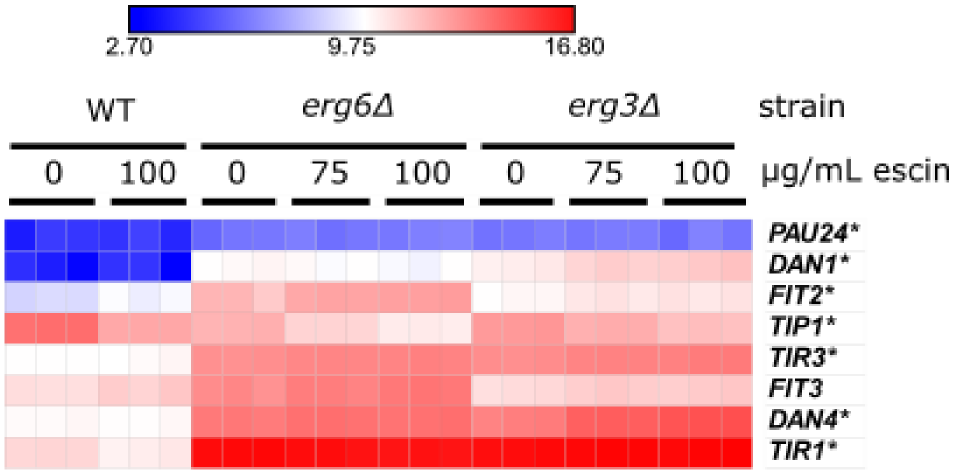

Figure S8: Expression of differentially expressed cell wall mannoprotein genes
Expression of mannoprotein genes which are upregulated ≥2-fold in the comparison *erg6Δ* vs. WT (0 µg/mL escin). Asterisk; upregulated ≥2-fold in the comparisons *erg3Δ* vs. WT (0 µg/mL escin). Scale log2(CPM+4). Comparison FDR ≤0.1.

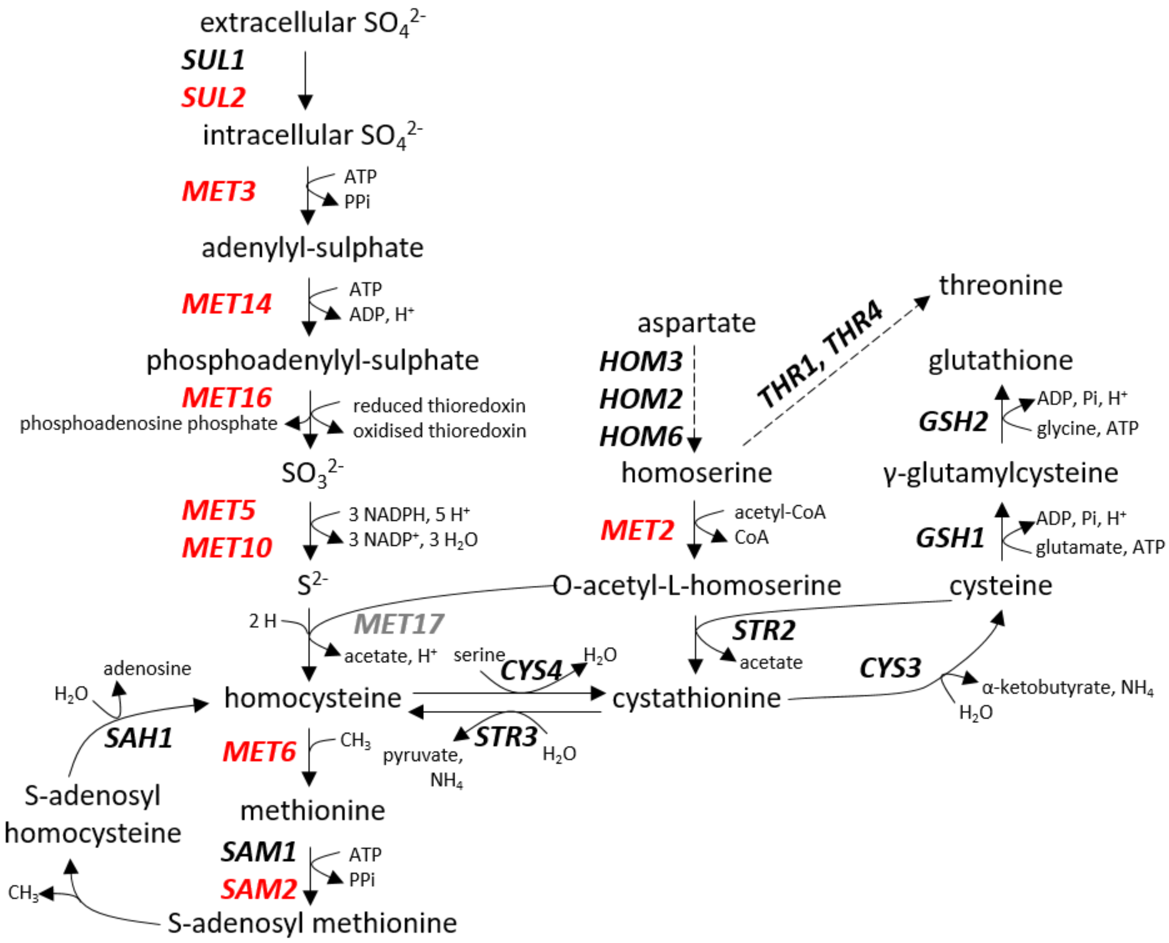

Figure S9: Overview of the methionine biosynthesis pathway. Red; gene upregulated ≥2-fold in *erg6Δ* 0 µg/mL escin versus WT 0 µg/mL escin (FDR ≤0.1). Dashed arrow; summary of multiple biosynthesis steps. The *MET17* gene (alias *MET15*) is disrupted in BY4741.

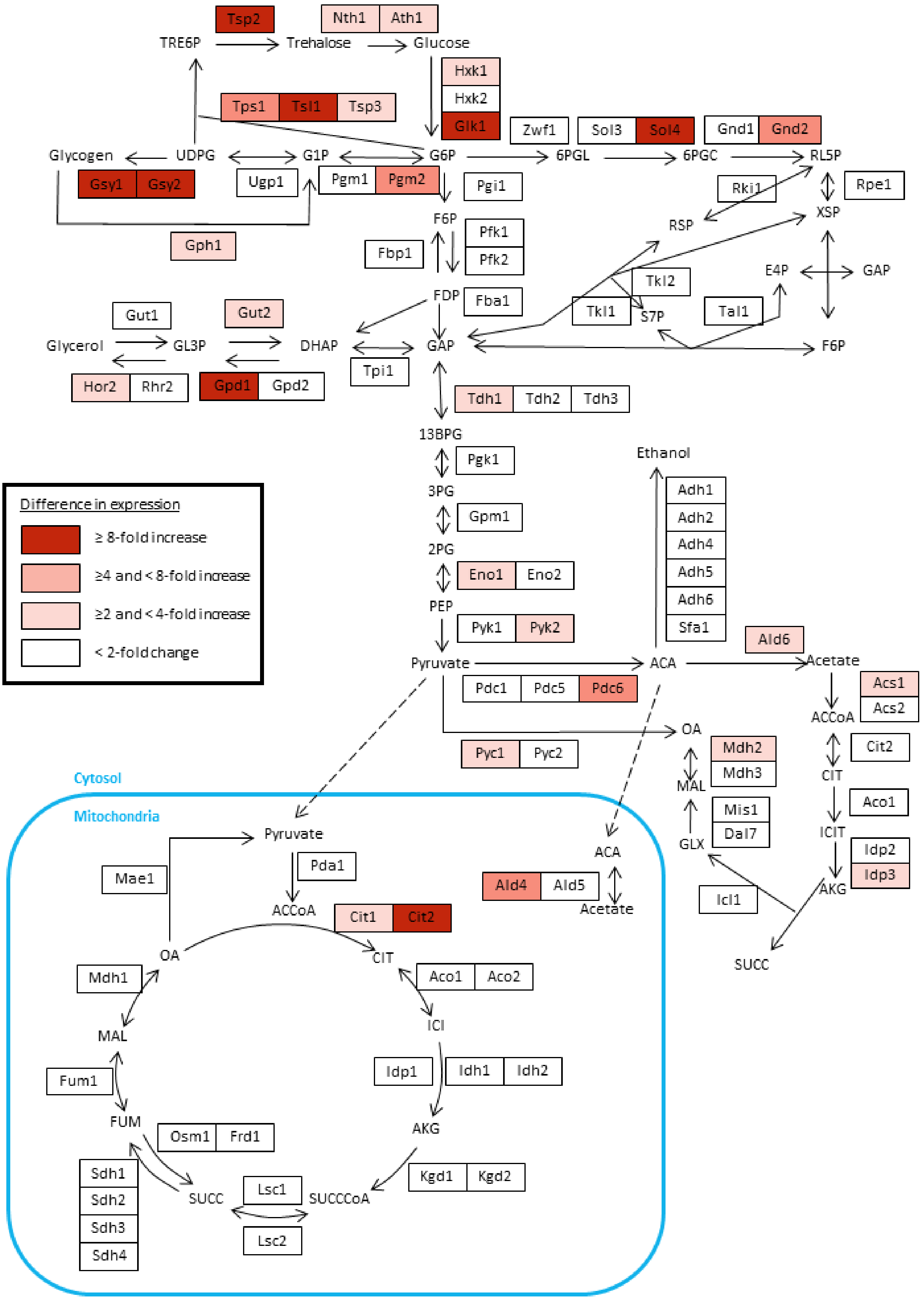

Figure S10: Impact of escin treatment on yeast central carbon metabolism

Overview of yeast central carbon metabolism, adapted from previously published diagrams[8,9]. Colour indicates difference in expression between WT 100 and 0 µg/mL escin treatments. None of the depicted genes are downregulated ≥2-fold in response to escin treatment.

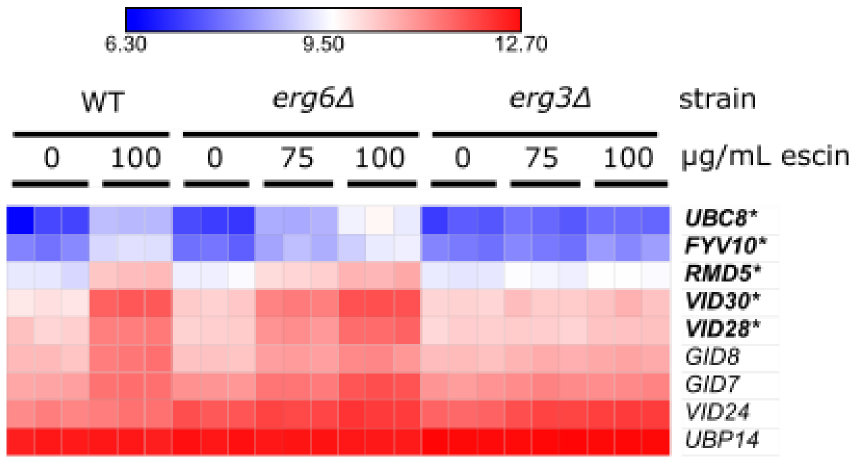

Figure S11: Expression of GID complex genes

Expression of genes encoding components of the GID complex[10], scale log2(CPM+4). Bold; upregulated ≥2-fold in the comparison WT 100 vs. 0 µg/mL escin. Asterisk; upregulated ≥2-fold in the comparisons *erg6Δ* 100 vs. 0 µg/mL escin. Comparison FDR ≤0.1.

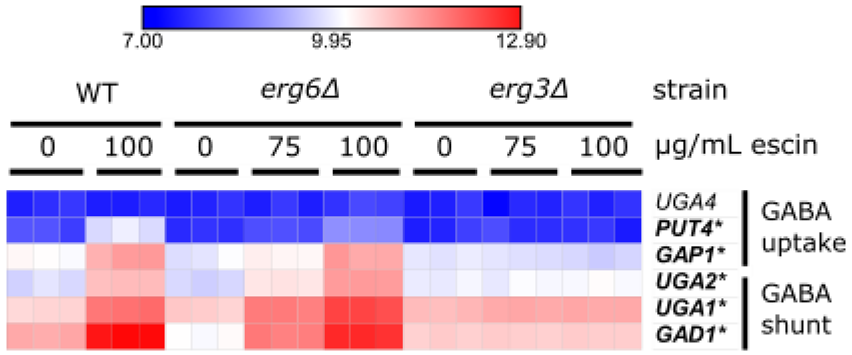

Figure S12: Expression of genes of the GABA shunt pathway

Expression of genes involved in γ-aminobutyric acid (GABA) uptake and the GABA shunt, scale log2(CPM+4). Bold; upregulated ≥2-fold in the comparison WT 100 vs. 0 µg/mL escin. Asterisk; upregulated ≥2-fold in the comparisons *erg6Δ* 100 vs. 0 µg/mL escin. Comparison FDR ≤0.1. *GAD1* encodes a glutamate decarboxylase, *UGA1* encodes a GABA transaminase and *UGA2* encodes a succinate semialdehyde dehydrogenase.

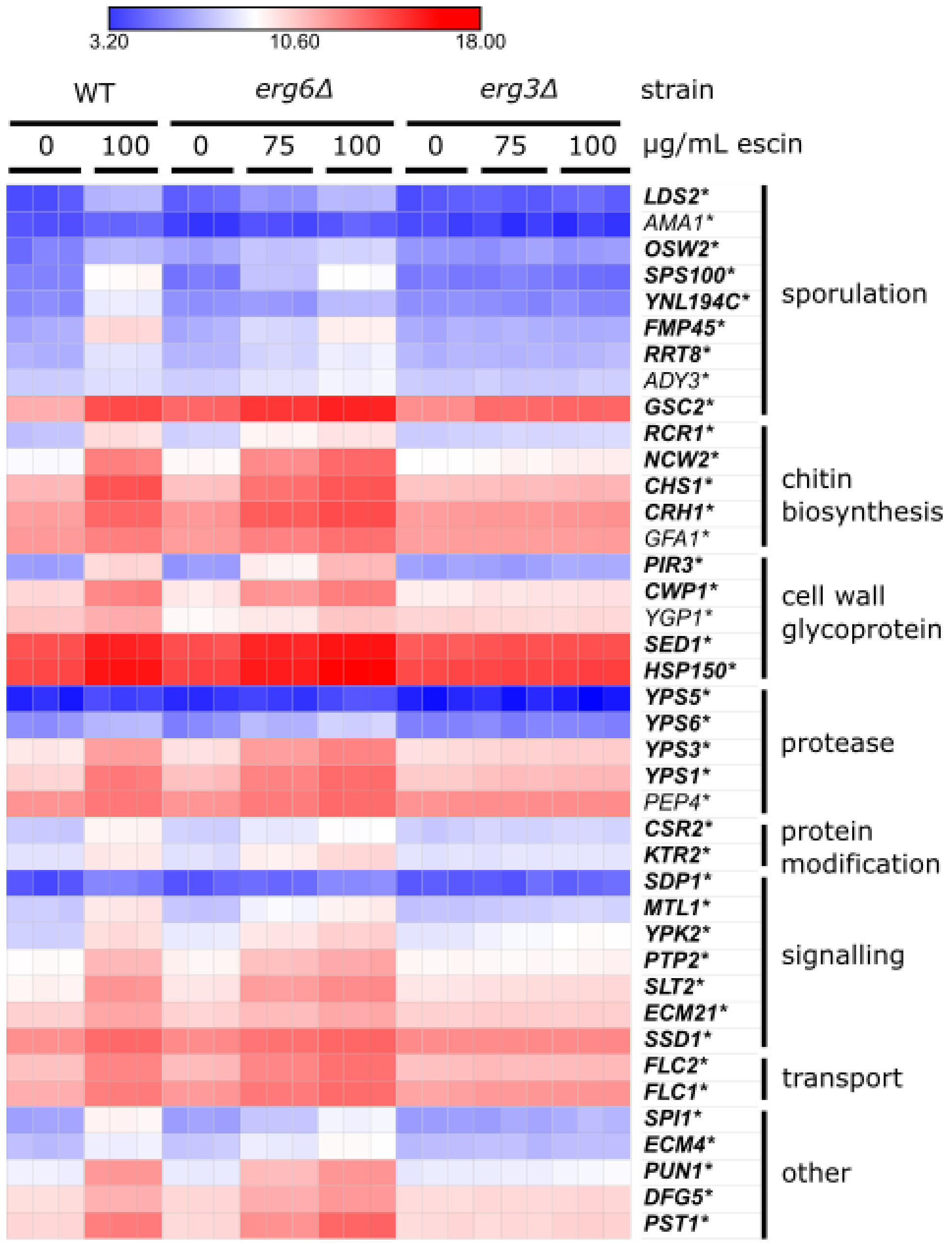

Figure S13: Escin response genes relating to cell wall organisation
Expression of genes relating to cell wall organisation which are upregulated ≥2-fold (FDR ≤0.1) in response to 100 µg/mL escin treatment in either BY4741 (bold) and/or *erg6Δ* (asterisk). Scale log2(CPM+4)

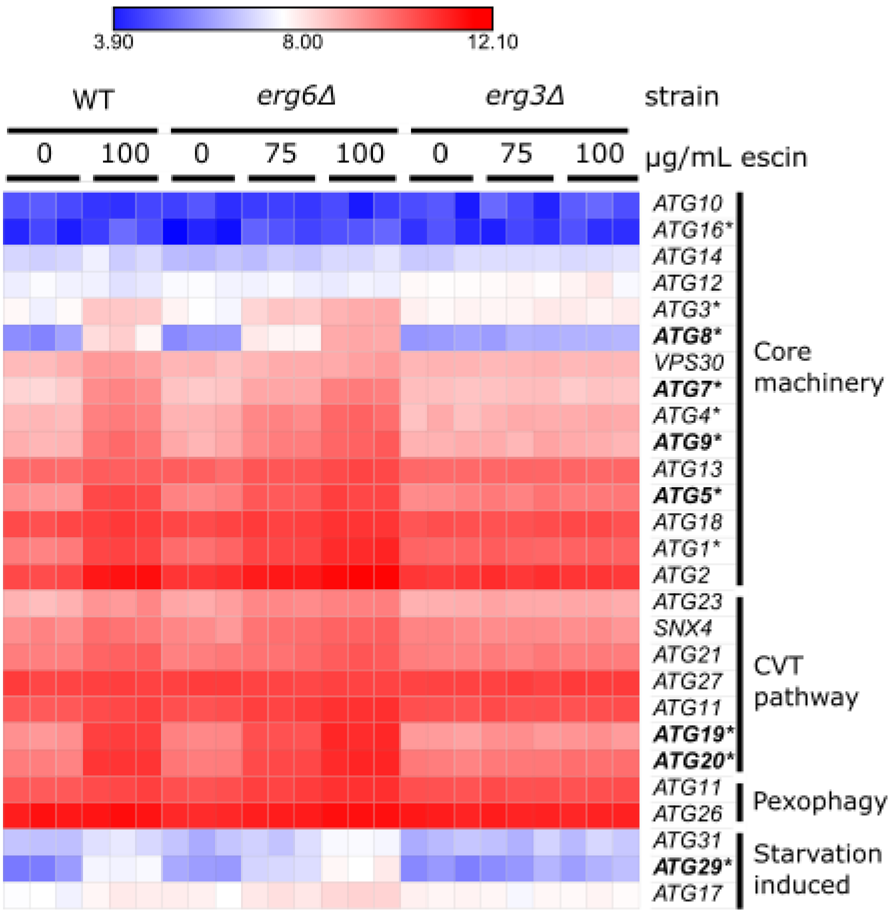

Figure S14: Expression of genes related to autophagy

Genes encoding proteins of the core machinery for membrane formation, the cytoplasm-to-vacuole (CVT) pathway, pexophagy and starvation induced autophagy[11]. The Atg11 protein is involved in both the CVT pathway and pexophagy. Expression scale log2(CPM+4). Bold; upregulated ≥2-fold in the comparison WT 100 vs. 0 µg/mL escin. Asterisk; upregulated ≥2-fold in the comparisons *erg6Δ* 100 vs. 0 µg/mL escin. Comparison FDR ≤0.1.

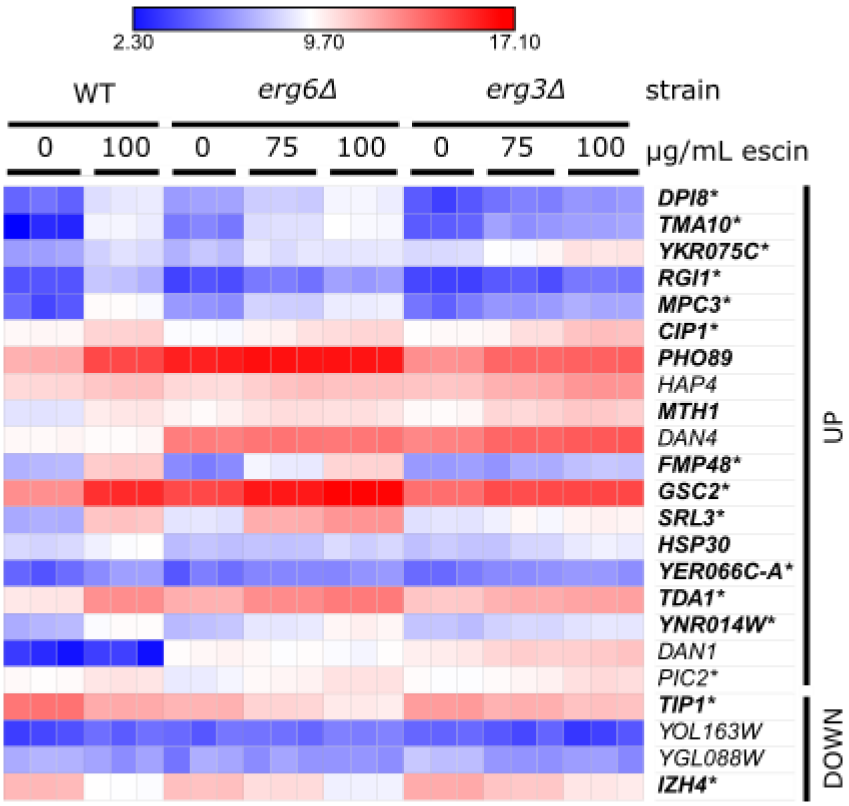

Figure S15: Expression of genes which are differentially regulated in the *erg3Δ* strain in response to escin treatment

Expression of genes which are up or downregulated ≥2-fold (FDR ≤0.1) in *erg3Δ* in the comparison 100 vs. 0 µg/mL escin, ordered from the largest to smallest log2 fold change. Expression scale log2(CPM+4). Bold; also differentially expressed ≥2-fold in the comparison WT 100 vs. 0 µg/mL escin. Asterisk; also differentially expressed ≥2-fold in the comparison *erg6Δ* 100 vs. 0 µg/mL escin.

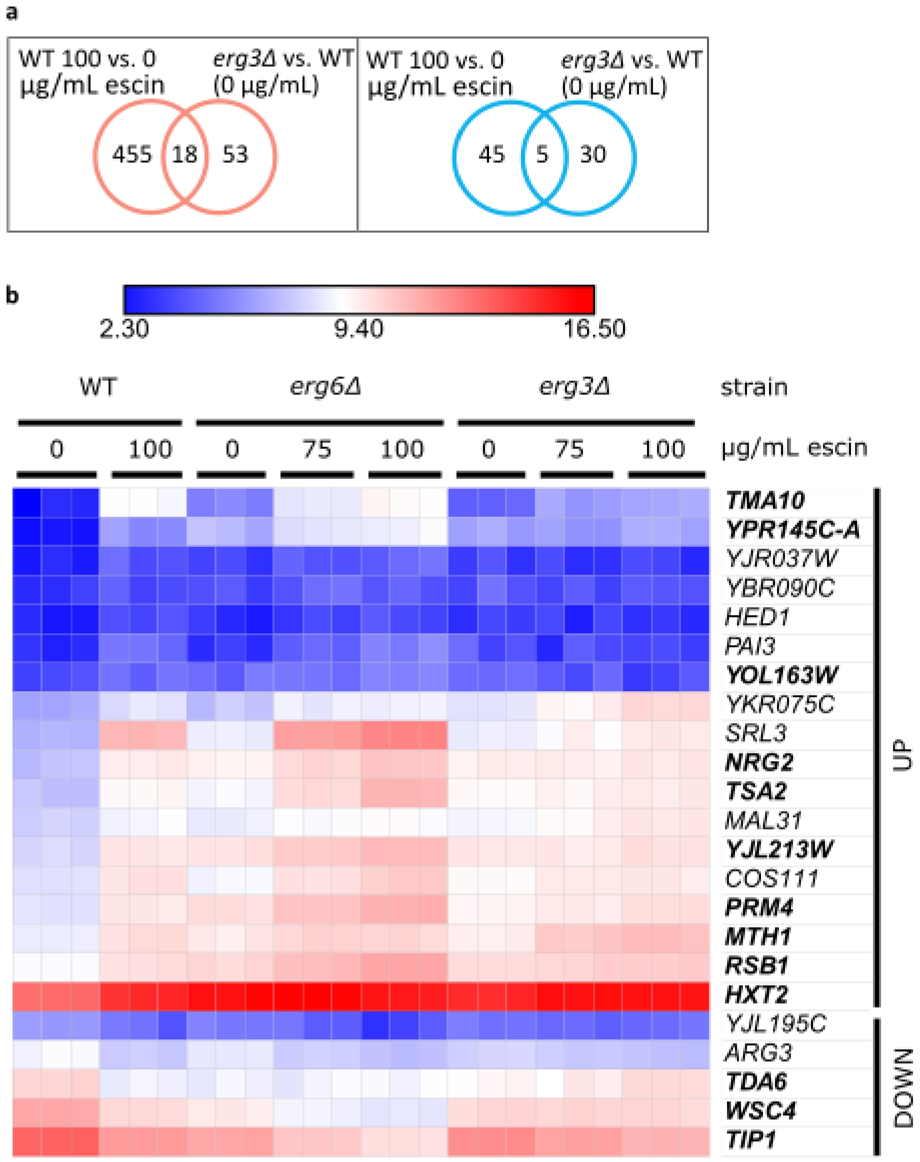

Figure S16: Overlap between escin response DEGs in the WT, and genes which are differentially expressed in *erg3Δ* under control conditions

a, Venn diagram for genes which are differentially expressed ≥2-fold in the comparison WT 100 vs. WT 0 µg/mL escin treatment, and the comparison *erg3Δ* 0 µg/mL vs. WT 0 µg/mL escin treatment (FDR ≤0.1). This comparison explores whether the *erg3Δ* strain could be ‘primed’ for the stress of escin treatment. **b**, Expression of the overlapping genes in (a), scale log2(CPM+4). Genes in bold are also differentially regulated ≥2-fold in the comparison *erg6Δ* 0 µg/mL vs. WT 0 µg/mL escin treatment.

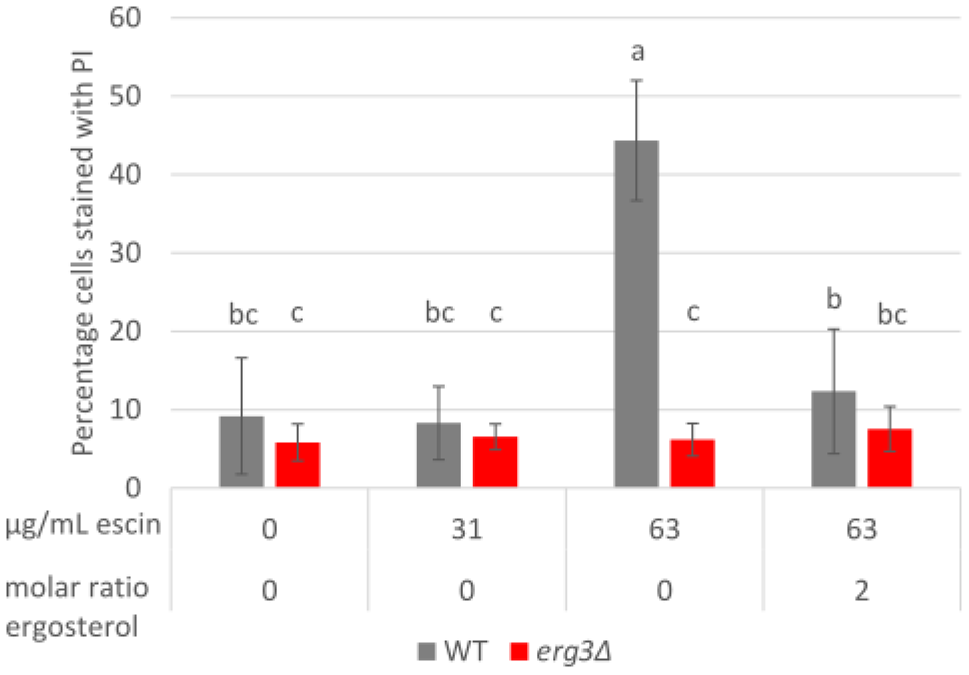

Figure S17: Staining of cells with propidium iodide
Mid-log cells were immobilised in chambered slides, treated with escin, escin:ergosterol mix, or control (1.25 % methanol) in PBS for 1 hour, washed, treated with propidium iodide for 20 minutes, washed, and then imaged. The average percentage of cells stained with PI is shown, ± SD for the 16 images analysed (4 biological replicates and 4 technical replicates). Statistics: one-way ANOVA with post-hoc Tukey HSD test, conditions not connected by the same letter are significantly different ($p \leq 0.05$).

References

1. Ge, S. X., Son, E. W. & Yao, R. iDEP: an integrated web application for differential expression and pathway analysis of RNA-Seq data. *BMC Bioinformatics* **19**, 534 (2018).
2. Ge, S. X., Jung, D. & Yao, R. ShinyGO: a graphical gene-set enrichment tool for animals and plants. *Bioinformatics* **36**, 2628–2629 (2020).
3. Heese-Peck, A. *et al.* Multiple Functions of Sterols in Yeast Endocytosis. *Mol. Biol. Cell* **13**, 2664–2680 (2002).
4. Guan, X. L. *et al.* Functional interactions between sphingolipids and sterols in biological membranes regulating cell physiology. *Mol. Biol. Cell* **20**, 2083–2095 (2009).
5. Bean, T. P. *et al.* Sterol content analysis suggests altered eburicol 14α-demethylase (CYP51) activity in isolates of Mycosphaerella graminicola adapted to azole fungicides. *FEMS Microbiol. Lett.* **296**, 266–273 (2009).
6. Martel, C. M. *et al.* Identification and Characterization of Four Azole-Resistant erg3 Mutants of Candida albicans. *Antimicrob. Agents Chemother.* **54**, 4527–4533 (2010).
7. Kenny, P. T. M. & Wetzel, J. M. Letter: Fragmentation Studies of Ergosterol. The Formation of the Fragment Ion at m/z 337. *Eur. Mass Spectrom.* **1**, 411–413 (1995).
8. Oliveira, A. P. *et al.* Regulation of yeast central metabolism by enzyme phosphorylation. *Mol. Syst. Biol.* **8**, 623 (2012).
9. Williams, T. C., Peng, B., Vickers, C. E. & Nielsen, L. K. The Saccharomyces cerevisiae pheromone-response is a metabolically active stationary phase for bio-production. *Metab. Eng. Commun.* **3**, 142–152 (2016).
10. Santt, O. *et al.* The Yeast GID Complex, a Novel Ubiquitin Ligase (E3) Involved in the Regulation of Carbohydrate Metabolism. *Mol. Biol. Cell* **19**, 3323–3333 (2008).
11. Nakatogawa, H., Suzuki, K., Kamada, Y. & Ohsumi, Y. Dynamics and diversity in autophagy mechanisms: lessons from yeast. *Nat. Rev. Mol. Cell Biol.* **10**, 458–467 (2009).
